# Lipocalin 2 Is a Regulator During Macrophage Polarization Induced by Soluble Worm Antigens

**DOI:** 10.3389/fcimb.2021.747135

**Published:** 2021-09-20

**Authors:** Hanyu Shen, Ziheng Wang, Ailong Huang, Dandan Zhu, Pingping Sun, Yinong Duan

**Affiliations:** ^1^Department of Pathogen Biology, School of Medicine, Nantong University, Nantong, China; ^2^Department of Clinical Biobank, Affiliated Hospital of Nantong University, Nantong, China

**Keywords:** schistosomiasis, bioinformatics, LCN2, macrophage, NF-κB

## Abstract

Caused by schistosomes, the human schistosomiasis is a tropical zoonotic parasitic disease. Pathologically, it occurs most often in the intestines and the liver, the sites of *Schistosoma japonicum* egg accumulation. The parasites’ produced eggs cause the main pathology in patients. Deposited parasite eggs in the liver induce the production of multiple cytokines that mediate the immune response, which in turn leads to granulomatous responses and liver fibrosis. These impact the hosts’ quality of life and health status, resulting in severe morbidity and even mortality. In this study, differentially expressed genes (DEGs) between ordinary samples and three 6- week infected mice were mined from microarray analysis based on the limma package. In total, we excavated the differential expression LCN2 was exhibited high expressions profile in GSE59276, GSE61376 demonstrated the result. Furthermore, CIBERSORT suggested detailed analysis of the immune subtype distribution pattern. *In vivo* experiments like real-time quantitative PCR, immunohistochemical (IHC) staining, and immunofluorescence (IF) demonstrated the expressions of LCN2 was significantly upregulated in *S. japonicum*–infected mice liver tissues and located in macrophages. Previous studies have shown that macrophages act as the first line of defense during schistosome infection and are an important part of liver granuloma. We used *S. japonicum* soluble worm antigens (SWA) to induce RAW264.7 cells to construct an *in vitro* inflammatory model. The current study aimed to investigate whether the NF-κB signaling network is involved in LCN2 upregulation induced by SWA and whether LCN2 can promote M1 polarization of macrophages under SWA treatment. Our research work suggests that LCN2 is significant in the development of early infection caused by *S. japonicum* and is of great value for further exploration. Collectively, the findings indicated that SWA promoted the expression of LCN2 and promoted M1 polarization of macrophages *via* the upregulation of NF-κB signaling pathway. Our findings demonstrate that NF-κB/LCN2 is necessary for migration and phagocytosis of M1 macrophages in response to SWA infection. Our study highlights the essential role of NF-κB/LCN2 in early innate immune response to infection.

## Introduction

A kind of digenetic trematode, schistosomes are zoonotic parasites that cause human schistosomiasis. On a global scale, approximately more than 240 million individuals from 76 nations have been afflicted with schistosomiasis (Lo et al., 2018). While several *schistosoma* species do exist, the primary pathogens against humans include *Schistosoma mansoni* (*S. mansoni*), *Schistosoma haematobium* (*S. haematobium*), and *Schistosoma japonicum* (*S. japonicum*) (Ross et al., 2013). *Schistosoma japonicum* is deemed the most dangerous among the three pathogens because of its greatest egg yield from female worms, as well as the potentially greatest life expectancy for adult worms (Chen, 2014). After infection, within three (3) weeks the schistosomes present inside the host body are depicted as the primary target of the immune defense. Around 2 weeks afterward, they start to emit eggs, which are then attached onto the host’s tissues to induce granulomatous reactions. By the 8^th^ week after infection, these reactions are at the highest point, consequently bringing severe symptoms to the host. By the 11^th^ to 13^th^ week, also known as the chronic infection stage, hepatic stellate cell (HSC) activation occurs, which in turn leads to the growing deposition of collagen in the liver of the host, resulting in the development of fibrosis (Pearce and MacDonald, 2002; Burke et al., 2009; Burke et al., 2011). The quality of health and life of the host is seriously impacted, resulting in acute morbidity and mortality also.

The macrophages are most essential innate cells that play various roles in host immune regulation and protection, schistosome-induced inflammation, and fibrosis. Previous studies have shown that in the acute stage of infection, Th1 cells play a pro-inflammatory role and can activate M1 macrophages, which elicit a microbicidal response that causes responses of anti-infection, pro-inflammation, and cell killing. The excessive polarization of M1 macrophages will aggravate tissue damage, but this pathological damage will be inhibited by IL-4 and IL-10 cytokines (Zheng et al., 2020). Th2 cells can induce M2 macrophage polarization during infection and play an anti-inflammatory, tissue repair, and fibrotic role. Its secreted IL-4 and IL-10 cytokines can aggravate the process of granuloma and the development of fibrosis. It will be significantly improved after treatment with anti-IL-4 neutralizing antibody (Fallon et al., 2000). During the process of *S. japonicum* infection, a large number of macrophages gather around the liver tissue during the acute granulomatous inflammation and hepatic fibrosis. Therefore, it is important to understand the molecular mechanism behind the macrophage polarization process underlying the *S. japonicum* infection processes (Burke et al., 2010).

Of late, high-throughput sequencing and *in-silico* development have paved the way for molecular innovation, bringing forth effective means to decode vital genetic or epigenetic changes for several aspects of medicine. From these innovative developments, molecular arrangements and systems and efficient biomarkers to address more illnesses have been determined. For instance, DNA microarrays, better known as gene chips, have rendered extensive and effectual realization toward profiling diverse diseases gene expressions. As a result, an all-encompassing and widespread public repository database known as Gene Expression Omnibus (GEO) has been promoted by the National Center for Biotechnology Information (NCBI). Furthermore, to settle any irregularity caused by heterogeneous trials or distinct detection policies, integrated bioinformatics analyses have been generally applied.

The 24p3, a secreted glycoprotein having a molecular weight of 25 kilodaltons (kDa), and Lipocalin 2 (LCN2) are also known as neutrophil gelatinase‐associated lipocalin (NGAL). LCN2 is expressed in cells such as neutrophils, macrophages, epithelial cells, adipocytes (Xiao et al., 2017). LCN2 is primally believed as a chief regulator of immune response. To efficiently diagnose inflammatory stimuli and induce successful cytokine responses, LCN2 was reported to be increased in macrophages. LCN2 potentiates the M1 phenotype of microglia, which is a chemokine inducer in the CNS (Jang et al., 2013). Previous studies have shown that the deficiency of LCN2 might hinder the usual expression of inflammatory factors secreted by macrophages after stimulation of *E. coli* O157:H7 (Wang et al., 2019). SWA is one of the major schistosome antigen mixtures and participates in the primary soluble proteins associated with the *S. japonicum* infection-induced adaptive immune response (Yang et al., 2017). SWA exclusively persuades the macrophages to M1 type cells. In spite of these recent studies, the understanding of LCN2 expression with its relationship to molecular mechanism in an early stage of schistosome infection remains largely unclear.

For this research, microarray analysis was conducted on the analysis of differentially expressed genes (DEGs) between ordinary samples and those obtained from three distinct mice for a 6-week group. The GSE61376 database was further used for validation on human chronic hepatic *S. japonicum*, and quantitative real‐time PCR (qRT-PCR) assay and IHC were further utilized to verify the distinctions in mice liver tissues following infection. Eventually, LCN2 was discovered, which can further determine the manifestation and development of *S. japonicum*, as well as the dysregulated pathways that may be engaged toward developed liver injury risk because of *S. japonicum* infection. Furthermore, we constructed the inflammation model to explore the molecular mechanism, and we found that SWA promoted the initiation of the NF-κB signaling pathway and induced the increase of LCN2 expression, which makes macrophage M1 phenotype. Workflow of this work has been submitted in **Supplementary Figure 1**.

## Materials and Methods

### Dataset Source and Preprocessing

To investigate the differential gene expression between *S. japonicum* infection samples and normal samples, the gene expression profiles of GSE61376 and GSE59276 were acquired and then assessed from the Gene Expression Omnibus (GEO) database (http://www.ncbi.nlm.nih.gov/geo/), an open database that documents high-throughput microarray empirical data (Edgar et al., 2002). These RNA profiles were provided on GPL6885 (Illumina MouseRef-8 v2.0 expression beadchip) and GPL6947 (Illumina HumanHT-12 V3.0 expression beadchip). GSE59276_RAW. tar and Series Matrix File were downloaded and classified *S. japonicum* infection groups and normal groups, and GSE61376 was downloaded to validate the hub genes.

### Identification of DEGs

Differential expression analyses are controlled by the limma package to conduct microarray and RNA-sequencing experiments. Excluding the genes with very low expression, in order to identify the DEGs between the normal and the *S. japonicum* infection groups (Ritchie et al., 2015), the significance analysis of the limma package was applied. P-value <0.01 and |logFC| > 2 were used as the cut-off criteria to select the significant DEGs. The genes with logFC > 2 were thought to be upregulated genes, and those with logFC < −2 were regarded as downregulated genes. Then we validated differences between human liver LCN2 expression for chronic patients with *S. japonicum* infections and people without history or indicators of schistosomiasis *via* the GSE61376 dataset.

### Analysis of Immune-Infiltrating cells

The evaluation of the qualified sizes of the 22 kinds of infiltrating immune cells was done through the CIBERSORT deconvolution, which included the Neutrophils and Eosinophils, Mast cells activated, Mast cell resting, Dendritic cells activated, Dendritic cells resting, Macrophages M0, Macrophages M1, Monocytes, NK cells resting, NK cell activated, T cells CD8, T cells gamma delta, T cells regulatory (Tregs), T cells follicular helper (Tfhs), T cells CD4 memory activated and resting, T cells CD4 naive, and B cells memory, and naive gene expression matrix was utilized (Newman et al., 2015). For establishing the gene expression datasets, the standard annotation files and the default signature matrix at 1,000 permutations were used.

### Animal Models

Six-week-old wild C57BL/6 mice subjects, with weights ranging between 18 and 22 g, were produced from Nantong University’s Laboratory Animal Center. Snails *Oncomelania hupensis* infected by nature were the sources of *S. japonicum* cercariae and were acquired from the Jiangsu Institute of Parasitic Disease in Wuxi, China. Similarly, to generate the models that were infested by *S. japonicum*, the subjects were infected through their skins with 15 ± 2 *S. japonicum* cercariae; afterward, at 0, 3, 6, 12 weeks, they were euthanized (n = 3 for each group, and every experiment was performed three times with similar results). Four groups were created after dividing the subjects, whilst the mice (C57BL/6) were casually separated into infected and normal groups. Animal precautions and experimental processes were sanctioned by Nantong University’s Animal Ethics Committee.

### Immunofluorescence

Paraffin was used for embedding the liver specimens, which were then sliced into 3 um thickness of tissue sections. Correspondingly, antibodies of F4/80+ and LCN2 macrophages were diluted by 200-fold. For 30 min tissues were blocked with 5% BSA, and then overnight the primary antibodies were incubated at 4°C. PBS was used for washing tissue sections three times followed by incubation with secondary Alexa Fluor^®^ 555 conjugate antibody (Invitrogen, USA) and Alexa Fluor^®^ 488 conjugate antibody (Jackson Immuno Research Laboratory, USA) for 1 h at 37°C. Once again the washing process was repeated with PBS for three times, nuclei were stained using DAPI, and fluorescence analysis was performed *via* a confocal laser scanning microscope.

### Immunohistochemical Staining

The liver tissues of the mice were cut into sections (4 µm thick) after embedding them with paraffin. For granuloma analysis, liver sections were stained and dewaxed with hematoxylin and eosin (H&E). Using a graded ethanol series, the sections were rehydrated after their dewaxing in xylene. Overnight, with the help of primary antibody, the sections were incubated at 4°C, after being washed with PBS. After being washed with PBS three times, and using HRP-conjugated goat anti-human Fab antibody, the sections were incubated at room temperature for 30 min and washed with PBS three times. The color was developed with diaminobenzene (DAB) solution. Finally, to counterstain the sections, hematoxylin was used. Under the microscope, the slides were examined after normal washing, dehydration, and lucidification. The positively stained cells were identified as the cells stained brown.

### Cell Culture and Treatment

Mouse monocyte/macrophage RAW264.7 cells (Cell bank of Chinese Academy of Sciences, Shanghai, China) were cultured in DMEM that contained 10% fetal bovine serum (FBS) and put within a humidified incubator with 5% CO_2_ at 37°C. SWA and SEA were obtained from the Jiangsu Institute of Parasitic Diseases. In the present study, RAW264.7 cells at a density of 1 × 10^6^ cells/well were plated in six-well plates and cultured for 12 h. Then SWA was added at various concentrations for 24 h.

### Small Interfering RNA Transfection

For the transfection of small interfering RNA (siRNA), RAW264.7 cells were plated to a six‐well plate at a density of 2 × 10^5^ cells per well. Similarly, the transfection of LCN2, siRNA, and scrambled‐siRNA (GenePharma, Shanghai, China) was performed by INTERFERin^®^ transfection reagent (Polyplus-transfection, Illkirch, France) following the manufacturer’s protocol. Furthermore, Scrambled‐siRNA was used as a control for non-sequence‐specific effects. Temporarily, INTERFERin^®^ transfection reagent (10 μl) was combined with 5 μl siRNA in a total of 400 μl DMEM. The whole medium was added to each well after 6 h of incubation. RAW264.7 cells were additionally incubated for 24–48 h.

### RNA Extraction, Reverse Transcription, and qRT-PCR

Using the RevertAid First-Strand cDNA Synthesis Kit (Thermo Fisher Scientific, USA), the reverse-transcribed to cDNA, and the Trizol RNA isolation reagent (Invitrogen, USA), the total RNA was extracted from the liver of the mouse. Using the SYBR Premix Ex Taq Kit (Takara, Japan) with specific primers for target genes on a StepOnePlus Real-Time PCR System (Applied Biosystems, USA), the qRT-PCR was performed. In the same samples, the levels of expression of all the transcripts were normalized to GAPDH. Lastly, the cycling parameters were as follows: 40 cycles of 95°C for 5 s and 62°C for 30 s, and 72°C for 30 s. Using the comparative CT method (2^−ΔΔCt^), relative expression levels were calculated. Gene-specific primer sequences were as follows: GAPDH, forward (5′-TGGAAAGCTGTGGCGTGAT -3′) and reverse (5′-TGCTTCACCACCTTCTTGAT-3′); LCN2, forward (5′-ACC ACG GAC TAC AAC CAG TTC GCC-3′) and reverse (5′-ACT TGG CAA AGC GGG TGA AAC G-3′); INOS, forward (5′-GTTCTCAGCCCAACAATACAAGA-3′) and reverse (5′- GTGGACGGGTCGATGTCAC -3′) (Kong et al., 2018); IL-6, forward (5′- GAGGATACCACTCCCAACAGACC -3′) and reverse (5′- AAGTGCATCATCGTTGTTCATACA -3′); ARG1, forward (5′- CTCCAAGCCAAAGTCCTTAGAG -3′) and reverse (5′- AGGAGCTGTCATTAGGGACATC -3′); IL-4, forward (5′- ATGGGTCTCACCTCCCAACTG -3′) and reverse (5′- TCAGCTCGAACACTTTGAATAT -3′) (Bai et al., 2020).

### Western Blotting

RAW264.7 cells pretreated with or without SWA for 24 h were harvested and resuspended in protein lysis buffer to extract protein. Then, SDS-polyacrylamide gels of 10% were used for loading the lysates and were transferred to a polyvinylidene difluoride (PVDF) membrane. Then, it was incubated with primary antibodies for LCN2 (1:1,000, Abcam, Cambridge, MA, USA); NF-κBp65 (1:500, Santa Cruz Biotechnology, Santa Cruz, CA, USA); phospho-NF-κBp65, IκBα, phospho- IκBα (1:1,000, Cell Signaling Technology, Danvers, MA, USA) overnight. We detected the primary antibodies with HRP‐conjugated secondary antibody at room temperature for 2 h. Following the manufacturer’s instructions, we visualized the proteins by enhanced chemiluminescence kit (Merck). Finally, the protein bands were normalized to GAPDH (1:1,000, Beyotime, China), and expression of the protein was quantified by Image J (National Institutes of Health, MD, USA).

### Statistical Analysis

R software and Perl language were utilized to perform bioinformatics analysis based on GEO datasets. To conduct difference comparisons of two groups, Student’s t-test was used, and to conduct difference comparisons of more than two groups, one‐way ANOVA was used. The GraphPad Prism 8 software (GraphPad, CA, USA) was used for analyzing the statistical significance of differences in experimental groups. We set our statistical significance for differences with p-value <0.05 (*p<0.05, **p<0.01, ***p<0.001).

## Results

### Gene Expression Profiles in *S. japonicum* Infection

**Figure 1:**
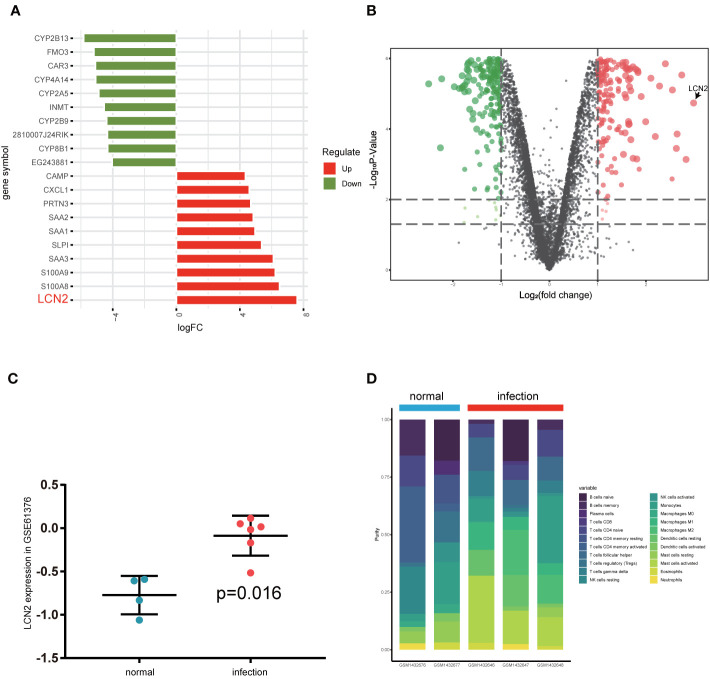
Investigation of differentially expressed gene analysis *via* GSE59276 datasets. **(A)** Top 10 up and down DEGs from GSE59276 datasets. **(B)** The volcano plot shows that LCN2 is the most significant gene. **(C)** The expression level of LCN2 between human normal tissues and infected tissues in GSE61376 dataset. **(D)** Relative proportion of 22 types of infiltrated immune cells in normal and infection groups.

After gene expression, profile data processing, and standardization, we integrated bioinformatics analysis for screening DEGs in GSE59276 dataset. Analyzed with the limma package, we classified a total of 222 overlap DEGs between normal and *S. japonicum* extracted from the GSE59276 datasets. We identified 222 DEGs *via* the limma package, with p < 0.01 and |logFC| > 2 cutoff criteria, and 141 of these DEGs showed significant upregulated, and the remaining 81 presented downregulated (**Supplementary Table 1**). One of the top genes presenting upregulated expression in the *S. japonicum* infected group was LCN2 (**Figures 1A, B**). Then we validated differences between human liver hub gene expressions for chronic patients with *S. japonicum* infections and people without history or indicators of schistosomiasis *via* GSE61376 dataset (**Figure 1C**). In the current study, we focused on the LCN2 gene and explored the possible mechanisms because it upregulated in patients with *S. japonicum* infections.

### Analysis of Immune-Infiltrating Cells

CIBERSORT algorithm was used to analyze the difference of immune-infiltrating cell components in the mice liver infected with *S. japonicum.* **Figure 1D** and **Supplementary Table 2** showed relative proportion of 22 types of infiltrated immune cells in normal and infection groups. We found that macrophages increased significantly in schistosomiasis group and accounted for the main components. Due to the limited number of samples, the infiltration of immune cells in the liver after schistosome infection is not fully revealed. The outcomes of the study suggested that macrophages in the early stage of *S. japonicum* infection have an important role.

### *S. japonicum* Infection Increases the LCN2 Expression Secreted by Macrophages in Mice Liver

**Figure 2:**
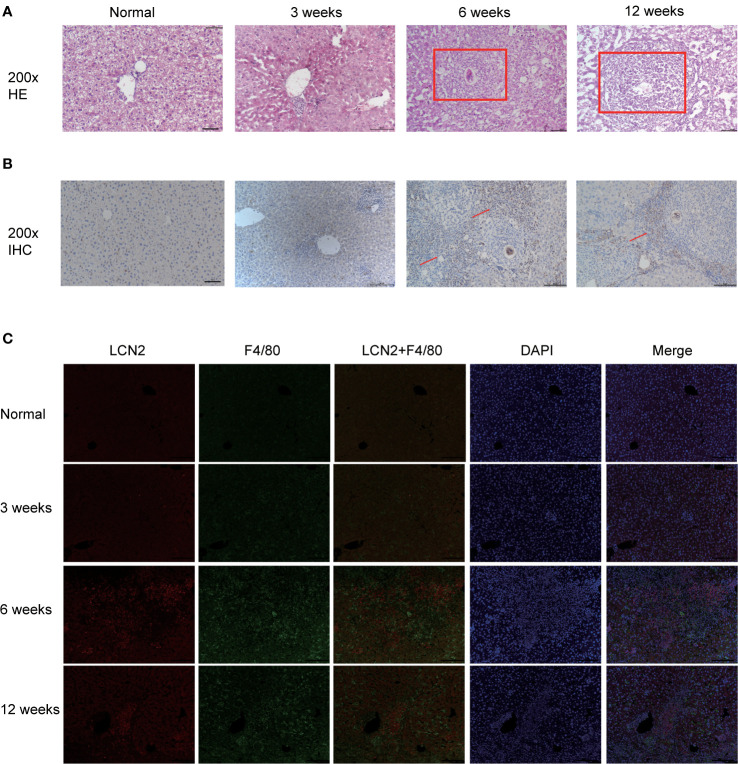
Validation of LCN2 expression levels *in vivo* (0, 3, 6, 12 weeks). **(A)** H&E staining showed that the diameter of single granuloma at 6 weeks after infection was significantly larger than that at 12 weeks. **(B)** The expression of LCN2 was significantly increased by immunohistochemistry. **(C)** LCN2 was co-localized with macrophages and expressed significantly at 6 weeks.

**Figure 3:**
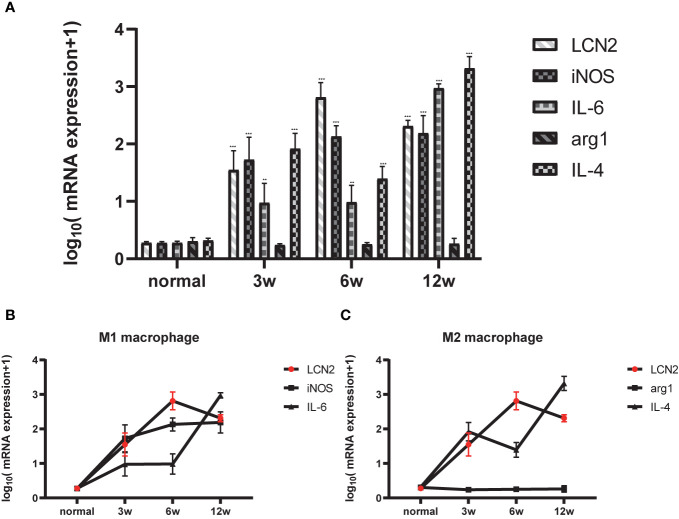
Relationship between LCN2 and macrophage polarization phenotype. **(A)** Total RNA was extracted from the liver tissues of normal and infected mice for 0, 3, 6, 12 weeks. [(*p < 0.05, **p < 0.01, ***p < 0.001)] **(B)** M1 polarized molecule iNOS began to peak at 3 weeks and reached the most significance at 6 weeks, lcn2 showed the same trend. **(C)** M2 polarized molecule IL-4 began to peak at 3 weeks and reached the most significance at 12 weeks.

Next, we constructed a model of *Schistosomiasis japonica* infection by infecting cercariae. Liver sections were embedded in paraffin and then stained using hematoxylin and eosin (H&E), original magnification: ×200 (**Figure 2A**). Then, to examine whether the expression of LCN2 in liver tissues is upregulated during infection, we evaluated the protein level of LCN2, which was performed in infected tissue samples and normal tissue samples from mice with *S. japonicum*. We found on the staining area and intensity revealing that LCN2 expression was increased in *S. japonicum*–induced inflammatory cell area of the necrotic-exudative granulomas (**Figure 2B**). As a consequence, LCN2 proteins were upregulated and investigated by IHC staining showing dark brown. In order to explore whether LCN2 is expressed in macrophages, we used immunofluorescence staining with F4/80 +, a marker of macrophage cells. The LCN2 signal was co-localized with macrophages demonstrated clearly from the immunofluorescence data (**Figure 2C**). The relative mRNA expression trend of macrophage genes and *Lcn2* using the qRT-PCR assay in the infected mice with *S. japonicum* was detected to explore the LCN2 response to radiation and to verify the accuracy of the microarray data analysis. As expected, the results showed that the average *Lcn2* mRNA expression level was significantly higher after infected 6 weeks in mice liver tissues compared with non-infected liver tissues, which were consistent with the results of the data analysis (**Figure 3A**). We also detected the changes of confirmatory factors in mice and drew a line diagram to observe the inflammatory molecules in mice (**Figures 3B**, **C**).

### SWA-Promoted LCN2 and Inflammatory Production in RAW264.7 Macrophages

**Figure 4:**
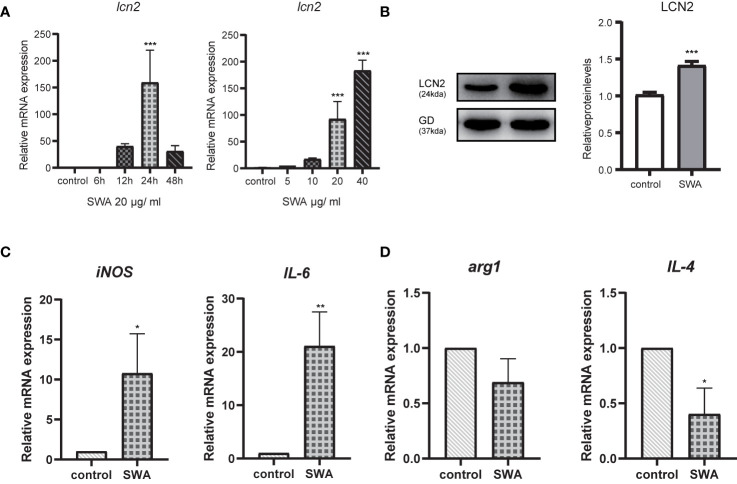
Soluble Worm Antigens (SWA) increases the secretion of LCN2 in macrophages RAW264.7 cells. **(A)** The secretion of LCN2 by RAW264.7 increased in a concentration-dependent manner. **(B)** LCN2 increased most significantly at 24 h after SWA stimulation. **(C, D)** After 24 h of SWA treatment, compared with the control group, M1 polarization increased significantly, while M2 polarization decreased (*p < 0.05, **p < 0.01, ***p < 0.001).

Previous studies have shown that mice infected with *S. japonicum* had the percentage of M1 macrophages significantly increased, and at the acute stage of *S. japonicum* infection, macrophages are typically skewed from the M1 phenotype toward the M2 phenotype (Zhu et al., 2014). Soluble egg antigen (SEA) and adult worm antigen (SWA) are the main soluble proteins that are targeted by the adaptive immune response induced by *S. japonicum* infection. We used these two antigens to stimulate RAW264.7 cells *in vitro* and found that LCN2 increased most significantly under the induction of SWA (**Supplementary Figure 2**). SWA stimulation significantly increased the percentage of M1 but not M2 macrophages. We next further explored the relationship between LCN2 and SWA in RAW264.7 macrophages. We treated RAW264.7 cells with SWA, at various times (0, 6, 12, 24, 48 h) and concentrations (0, 5, 10, 20, 40 μg/ml) (**Figures 4A, B**). The addition of SWA markedly increased *Lcn2* expression and secretion in a dose-dependent manner, and after 24 h of SWA treatment, the expression of *Lcn2* increased most significantly. Since the concentration of SWA in *S. japonicum* was less than 40 μg/ml, we treated macrophages with 20 μg/ml SWA for 24 h (**Figure 4C**). **Figure 4D** clearly shows that SWA treatment significantly increased the percentage of M1 but not M2 macrophages compared to control group. SWA treatment specially improved the expression of the M1 macrophage marker *IL6* and main enzyme of arginine metabolism *iNOS*. By contrast, SWA upregulated the M2 macrophage marker *IL-4* and the main enzyme of arginine metabolism *arg1*.

### LCN2 Deficiency Attenuates M1 Macrophages Polarization Under SWA Treatment

**Figure 5:**
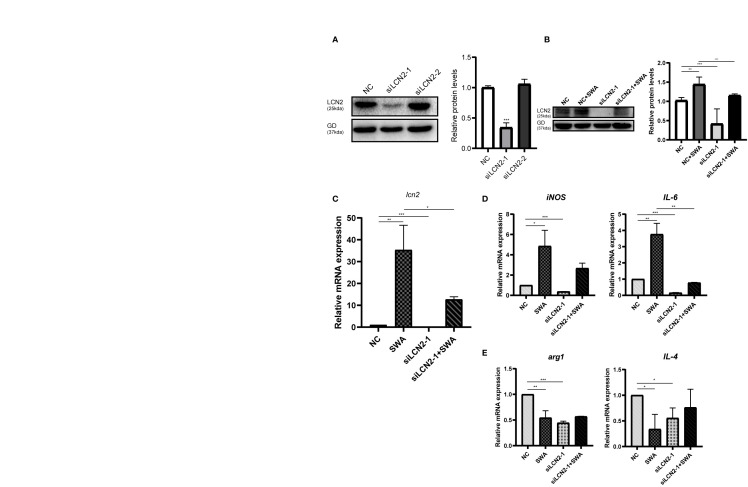
Inhibition of LCN2 expression reduced the effect of SWA on M1 polarization of macrophages. **(A)** Western blot analysis observed the knockdown efficiency LCN2. **(B, C)** Knockdown of LCN2 inhibits the upregulation of LCN2 induced by SWA. **(D)** Knockdown of LCN2 significantly inhibited the expression of M1 macrophage–related molecules. **(E)** Knockdown of LCN2 had no significant effect on M2 polarization. Statistical analyses were carried out by Student’s t-test. (*p < 0.05, **p < 0.01, ***p < 0.001).

The increased LCN2 may further regulate M1 macrophage polarization under SWA treatment. By knockdown of LCN2 in RAW264.7 cells (**Figures 5A, B**), we further verified the relationship between LCN2 and M1 macrophage. Transfection of LCN2 siRNA into RAW264.7 cells caused the obvious decrease in inflammatory cytokines (*IL-6* and *iNOS*) as evaluated by quantitative real‐time PCR analysis (**Figures 5C, D**). Consistent with expectations, the knockdown of LCN2 inhibited the upregulation of *IL-6* and *iNOS* induced by SWA. Meanwhile, the knockdown of LCN2 did not significantly affect M2 gene expression (**Figure 5E**). It indicated that SWA promotes M1 phenotype through upregulating LCN2 expression. Hence, our results showed that LCN2 played a vital role in macrophage stimulation by SWA and maintained their balances.

### NF-κB Signaling Pathway Is Necessary for SWA-Induced LCN2 Gene Expression

**Figure 6:**
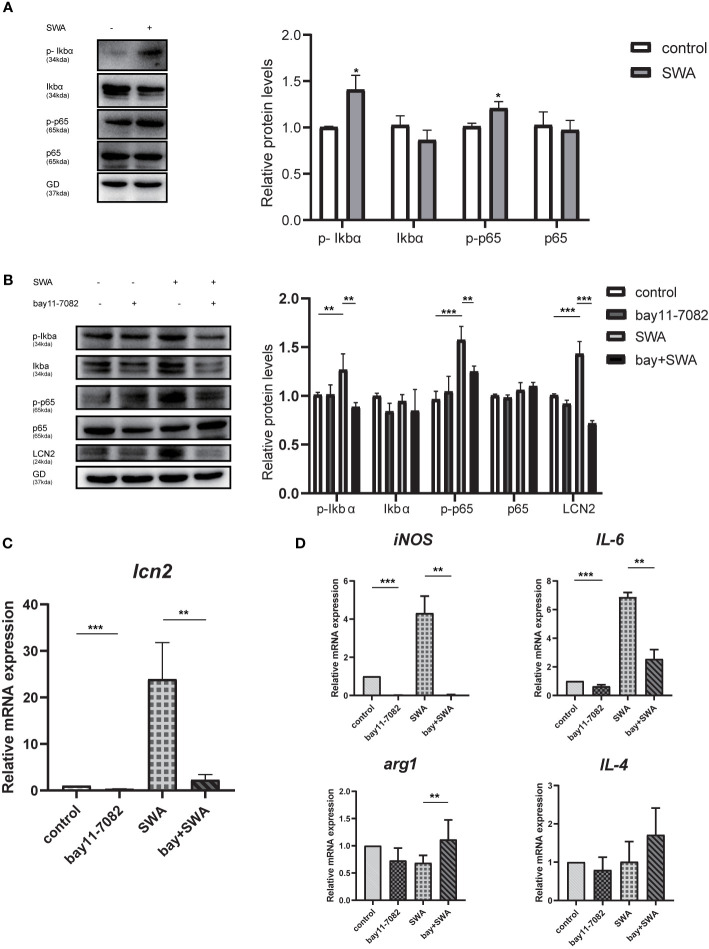
NF-κB signaling pathway upregulated the expression of LCN2 and affected the polarization of M1 macrophages. **(A)** SWA can activate NF-KB signaling pathway. Western blot analysis showed phospho-NF-κBp65, phospho-IκBα activation, and IκBα degradation. **(B)** NF-κB inhibitors Bay11-7082 can inhibit the expression of LCN2, phospho-NF-κBp65, and phospho-IκBα after SWA treatment. **(C, D)** Bay11-7082 inhibited LCN2 expression and M1 phenotype with or without SWA treatment (*p < 0.05, **p < 0.01, ***p < 0.001).

We explored how SWA induces LCN2 gene expression in the RAW264.7 cells. Previous study demonstrated TNFα requires NF-κB to induce LCN2 expression (Zhao et al., 2014). In other infections, such as mycoplasma infection, the expression of LCN2 in HC11 cells needs to be regulated by NF-κB (Zhao et al., 2020). Here, we explore whether SWA can induce LCN2 gene expression through NF-κB signaling pathway and whether SWA can affect macrophage polarization through NF-κB signaling pathway. Firstly, we found after SWA induction for 24 h, western blotting data demonstrated that Ikbα was significantly degraded and NF-kBp65 was phosphorylated, and NF-κB signaling pathway was active (**Figure 6A**). While discovering the potential anti-inflammation impact of NF-κB inhibitors in SWA treatment, RAW264.7 cell was treated with a panel of NF-κB inhibitors, Bay 11-7082. After 6 h of pretreatment and continue to culture for 24 h, bay 11-7082 effectively inhibited the increased expression of LCN2 caused by SWA and inhibited the activity of NF-κB pathway mediated by SWA (**Figure 6B**). After treatment with NF-κB inhibitor for 6 h, the expression of LCN2 decreased and the degree of M1 macrophage polarization downregulated after SWA stimulation. Therefore, we found that SWA could promote LCN2 expression and M1 polarization by activating NF-κB pathway (**Figures 6C, D**).

## Discussion

Schistosomiasis has an extreme effect on human health; in particular, *S. japonicum* serves as one of the primary pathogens of the said disease. Following the infection, eggs from the parasite are attached to the host liver tissues to provoke liver fibrosis and inflammation, resulting in irreparable liver damage and even death of the afflicted. Alongside a recently swift progress of microarray and high-throughput sequencing innovations, integrated bioinformatics procedures have been considerably operated to determine biomarkers that are associated with the identification, projection, and medication of several kinds of ailments. Here, we have found that LCN2 increased significantly in the early stage of schistosomiasis through preliminary biological information, then we explored the possible mechanism of increased LCN2 expression. From this research, the GSE59276 microarray datasets were designated to examine and compare DEGs between ordinary and *S. japonicum*–infected mice tissues. This research intended to identify what role LCN2 plays in schistosome infection. Through the above analysis, the integrated results were revealed. We used CIBERSORT deconvolution algorithm to find that macrophages increased significantly after *S. japonicum* infection.

Lipocalin 2 (LCN2), referred to as NGAL, is a twenty-five (25) kDa extensively analyzed secreted lipocalin protein that has the capability to transfer tiny lipophilic ligands. It is generated by several types of cells such as neutrophils, macrophages, adipocytes, and lymphocytes (Meheus et al., 1993; Flo et al., 2004; Wang et al., 2019). Over time, the LCN2 has been renowned as a promising entity in various pathological and physiological procedures, such as inflammation, iron homeostasis, organogenesis, microbial infection, tumorigenesis, and neurodegeneration (Liu et al., 2013; Buonafine et al., 2018). Previous studies demonstrated the expression of LCN2 can be induced by proinflammatory cytokines like LPS, IL-6, IL-17, IFN-γ, and it plays an important role in the pro-inflammatory reaction. Previous studies reported that in different inflammatory stimulation models, LCN2 can play different roles. Substantial increase in the expression of the pro-inflammatory factors was observed to be caused by the lipopolysaccharide (LPS) due to LCN2 deficiency in the murine inflammation model (Kang et al., 2018). Nonetheless, the absence of LCN2 may result in the high vulnerability of mice to *E. coli* O157:H7 infection while in decreased production of inflammatory cytokines when the bacteria *E. coli* 0157:H7 was encountered (Kang et al., 2018; Wang et al., 2019). During the current study, we examined the expression of LCN2 was significantly upregulated and that they could play a significant role in liver pathological changes caused by *S. japonicum*, such as acute liver granuloma and liver fibrosis, and we validated the difference between normal groups and schistosomiasis groups, then we investigated three pairs of infected subject liver tissues and the respective non-infected ones. Through the qPCR analysis and IHC staining, the same gene expression tendency was established as with the prementioned expression, hence validating the correctness of the research results.

In the current study, it was found that the expression of LCN2 increased in the liver of mice infected for 6 weeks, and LCN2 was secreted on macrophages. Macrophage is one of the main immune cells in the early stage of schistosome infection, which not only acts as innate immune cells to remove pathogens or apoptotic cells through phagocytosis but is also involved in the direct process of precise immune responses as antigen-presenting cells (Mosser and Edwards, 2008). Currently, it has been identified that macrophages maintain immune homeostasis significantly by changing the polarization of M1 subtype macrophages (Wynn et al., 2013). The M1 macrophages secrete pro-inflammatory cytokines and chemokines to stimulate inflammation in order to help in clearing the invaded pathogens. In the process of *S. japonicum* infection, M1 macrophages are the consequence of continuous response to injury (Barron and Wynn, 2011; Koyama and Brenner, 2017). Some literature has shown that the percentage of M1 macrophages in abdominal cavity after *S. japonicum* infection began to rise at 3 weeks after infection and began to decline at 8 weeks after infection. M2 macrophages were activated through m1-to-m2 phenotype transformation and began to rise from 8 weeks, but with different tissues, there may be differences in activated macrophages, which mainly depends on the changes of cytokines (Zhu et al., 2014; Song et al., 2020).

Hence, we constructed an *in vitro* model. *S. japonicum* worm antigens and egg antigens are the two main antigens secreted by *S. japonicum*, and the study showed that macrophages RAW264.7 cells were activated by SWA stimulation, resulting in M1 polarization. We found that SWA promoted macrophages to secrete LCN2, and the expression of LCN2 was upregulated in a concentration-dependent manner. The deficiency of LCN2 expression could induce macrophages to secrete inflammatory factors and inhibit macrophage M1 polarization. Then we explored the latent mechanism of SWA-induced increase of LCN2. Previous literature showed that the NF-kB pathway could activate macrophages to produce M1 polarization under LPS induction. NF-κB is the predominant transcription factor that activates inflammatory mediatory proteins such as cytokines, chemokines, and inducible enzymes (Liu et al., 2017). Literature has reported that NF-κB regulates LCN2 by binding to specific promoter sites during inflammatory stress. NF-κb can regulate the upregulation of LCN2 expression and stimulate inflammatory response in age-related macular degeneration (AMD)–induced inflammation (Zhao and Stephens, 2013; Ghosh et al., 2017). This may be one of the reasons for the increase of LCN2 expression in early infection, which depends on the activation of the NF-kB pathway. Our experiment confirmed that NF-kB can upregulate the expression of LCN2 and affect the polarization of M1 macrophages in the process of infection.

In conclusion, we found that LCN2 may play a vital role in schistosomiasis infection and revealed the possible molecular mechanism of LCN2. Fundamentally, an assembly of inflammatory cells such as macrophages, lymphocytes, neutrophils, and eosinophils was the most predominant cell type in the granulomas in *S. japonicum*. We found that LCN2 may be a positive regulator of macrophage M1 polarization and is closely related to cytokines. Macrophage M1 polarization may reduce liver fibrosis induced by *S. japonicum (* Souza et al., 2020). Our data revealed that the elevated LCN2 expression in *S. japonicum* infection might be a positive factor against it by upregulating INOS-mediated activation of macrophage M1. Our study found that SWA can promote the regulation of LCN2 and induce M1 polarization of macrophages. LCN2 is necessary in the process of macrophage polarization. The upregulation of LCN2 can be mediated by NF-κB signaling pathway. We hypothesized that LCN2 secretion may impact liver fibrosis. This needs to be proved by subsequent experiments. We will construct LCN2 knockout mice to observe the effect of LCN2 on liver damage, granulomas, and fibrosis induced by schistosomiasis infection.

## Data Availability Statement

The original contributions presented in the study are included in the article/**Supplementary Material**. Further inquiries can be directed to the corresponding author.

## Ethics Statement

The animal study was reviewed and approved by Nantong University(20180304-003).

## Author Contributions

HS participated in performing experiments, data visualization, writing original draft, and formal analysis. ZW participated in formal analysis and data curation. AH participated in performing experiments. DZ and PS participated in methodology and software. YD participated in conceptualization, resources, review and editing manuscript, supervision and funding acquisition. All authors contributed to the article and approved the submitted version.

## Funding

This study was funded by the National Natural Science Foundation of China (No. 81871677) and Postgraduate Research & Practice Innovation Program of Jiangsu Province (No. KYCX20_2839).

## Conflict of Interest

The authors declare that the research was conducted in the absence of any commercial or financial relationships that could be construed as a potential conflict of interest.

## Publisher’s Note

All claims expressed in this article are solely those of the authors and do not necessarily represent those of their affiliated organizations, or those of the publisher, the editors and the reviewers. Any product that may be evaluated in this article, or claim that may be made by its manufacturer, is not guaranteed or endorsed by the publisher.
