# Supplementary figures and images for: Lipocalin 2 Is a Regulator During Macrophage Polarization Induced by Soluble Worm Antigens
Source: Front Cell Infect Microbiol. 2021 Sep 20;11:747135. doi: 10.3389/fcimb.2021.747135 (PMC8489661; doi:10.3389/fcimb.2021.747135)

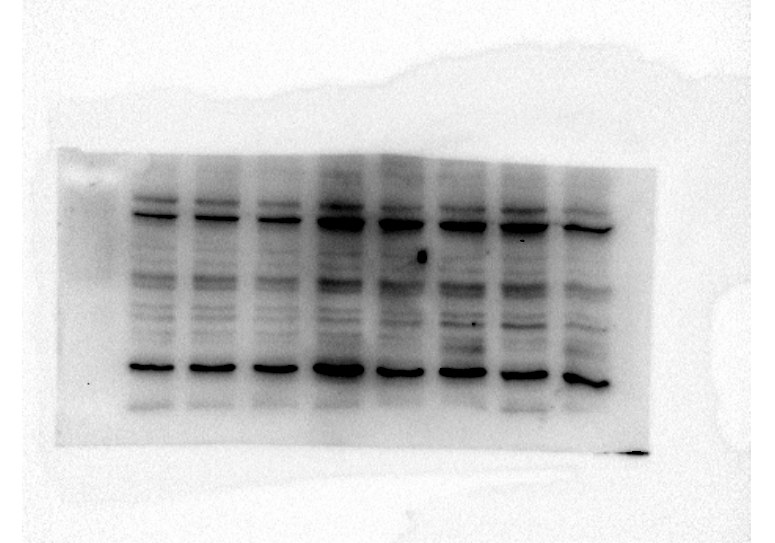

Supplement: Supplementary file 1 [file DataSheet_1.zip › WBs/4/Administrator 2021-01-30 14 -¦ 49 ++_Exposure_31_9sec.tif]

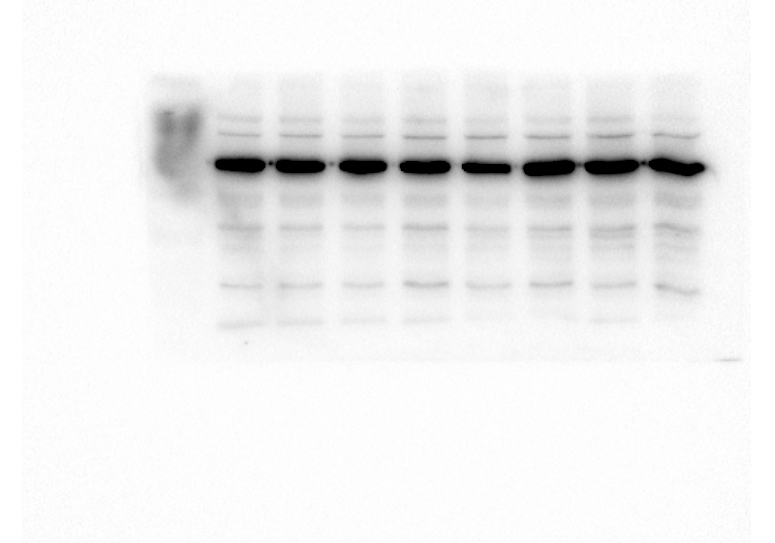

Supplement: Supplementary file 1 [file DataSheet_1.zip › WBs/4/Administrator 2021-01-31 12 -¦ 11 ++_Exposure_3_8sec.tif]

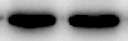

Supplement: Supplementary file 1 [file DataSheet_1.zip › WBs/4/GD.png]

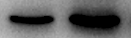

Supplement: Supplementary file 1 [file DataSheet_1.zip › WBs/4/LCN2-SWA.png]

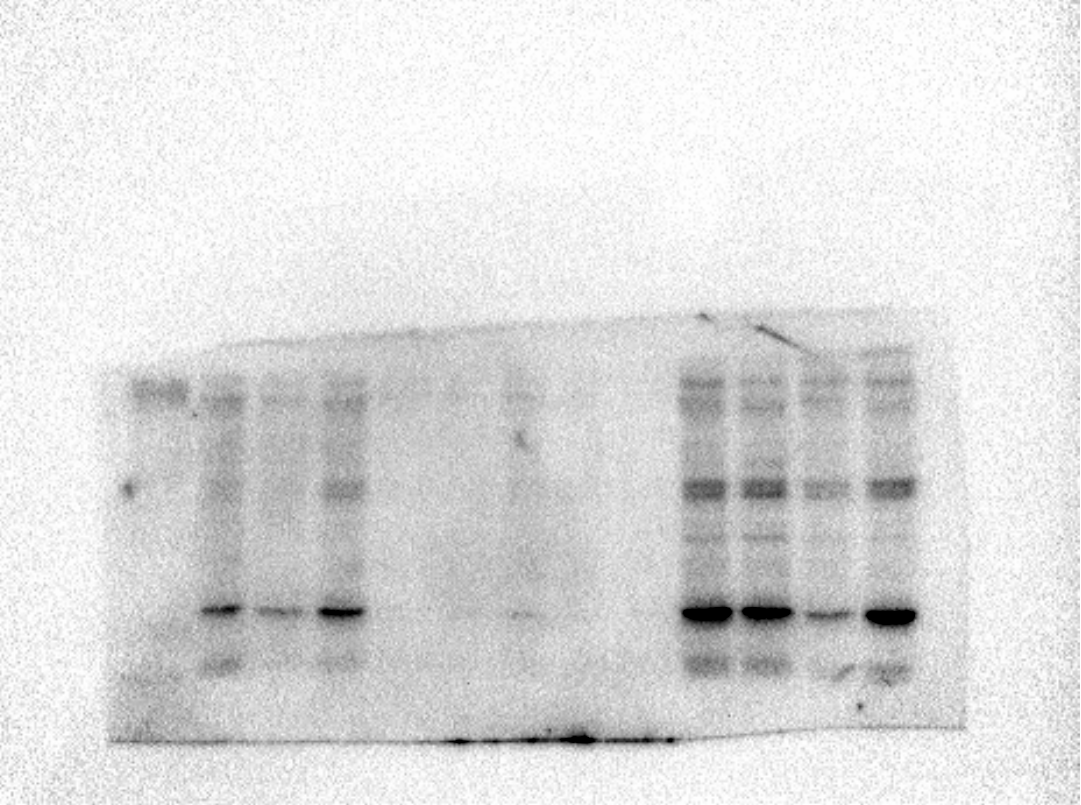

Supplement: Supplementary file 1 [file DataSheet_1.zip › WBs/5/Administrator 2021-03-18 15 -¦ 20 ++_Exposure_20_0sec.tif]

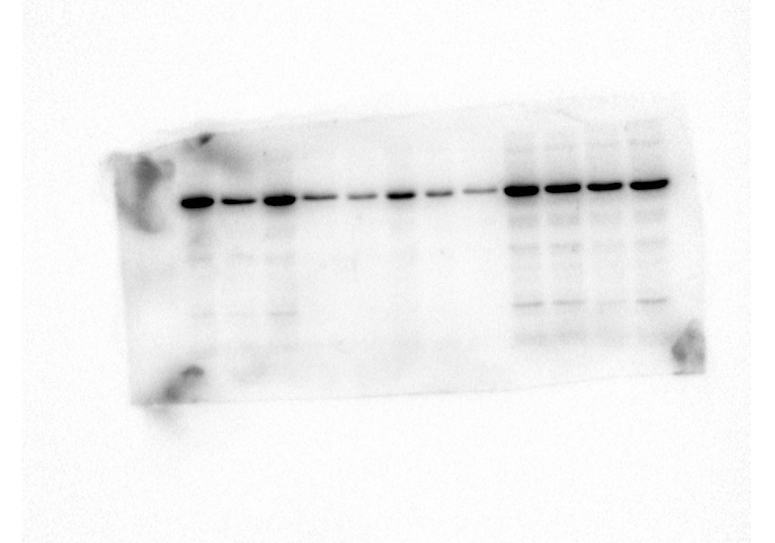

Supplement: Supplementary file 1 [file DataSheet_1.zip › WBs/5/Administrator 2021-03-19 12 -¦ 42 ++_Exposure_5_7sec.tif]

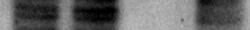

Supplement: Supplementary file 1 [file DataSheet_1.zip › WBs/5/LCN2.jpg]

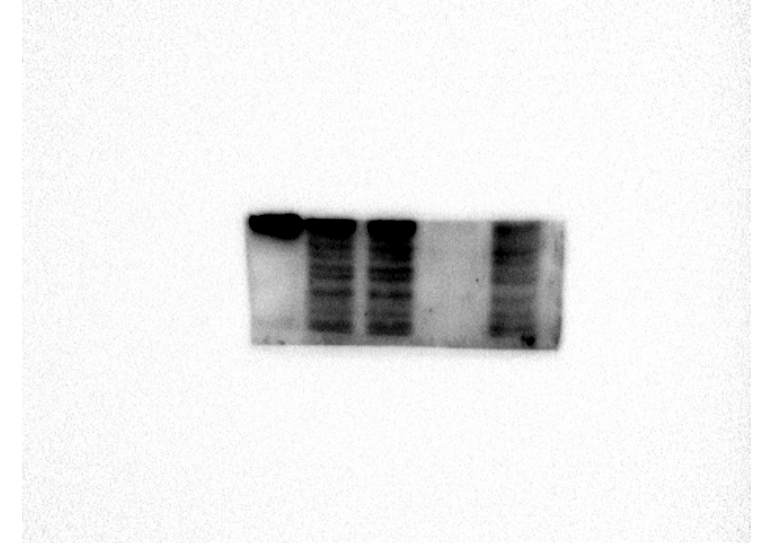

Supplement: Supplementary file 1 [file DataSheet_1.zip › WBs/5/LCN2.tif]

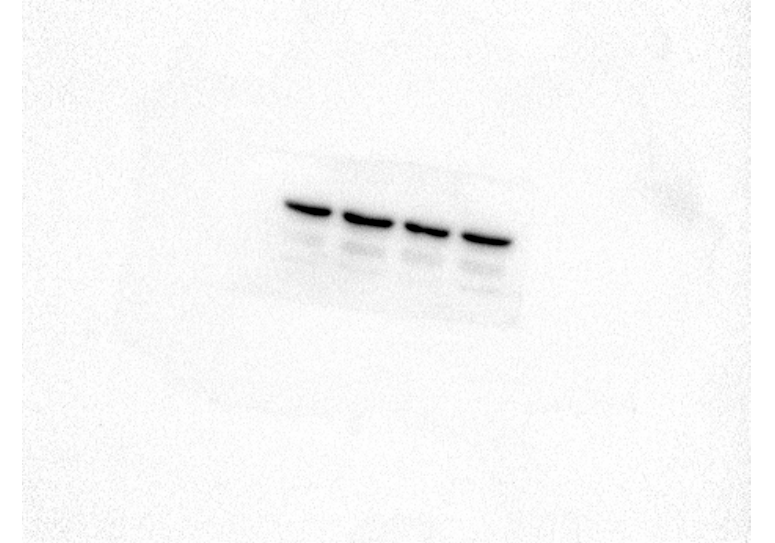

Supplement: Supplementary file 1 [file DataSheet_1.zip › WBs/6/6a/New folder/GD.tif]

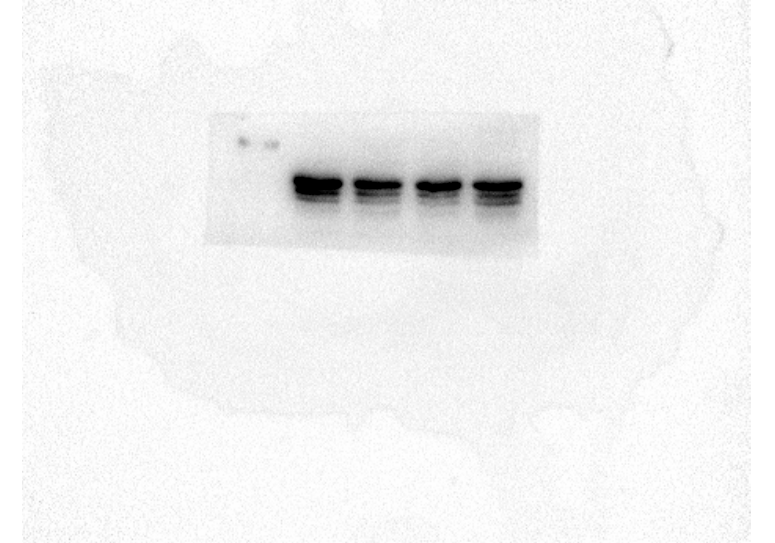

Supplement: Supplementary file 1 [file DataSheet_1.zip › WBs/6/6a/New folder/ikba.tif]

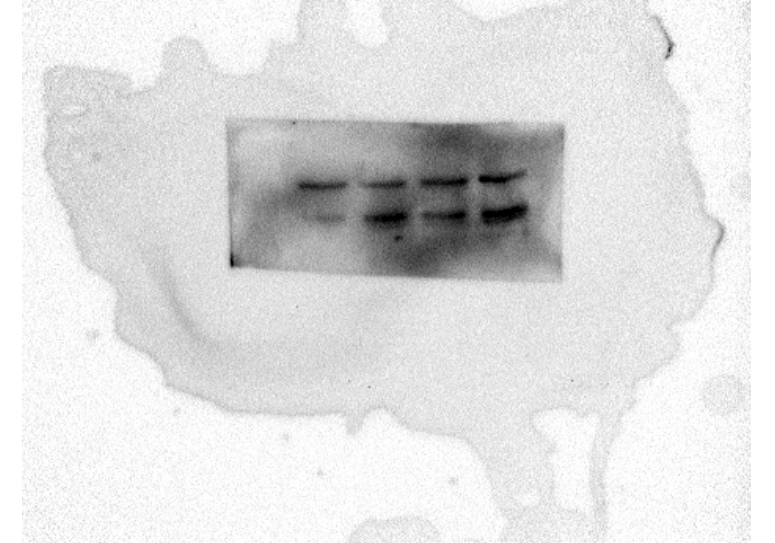

Supplement: Supplementary file 1 [file DataSheet_1.zip › WBs/6/6a/New folder/p-ikba.tif]

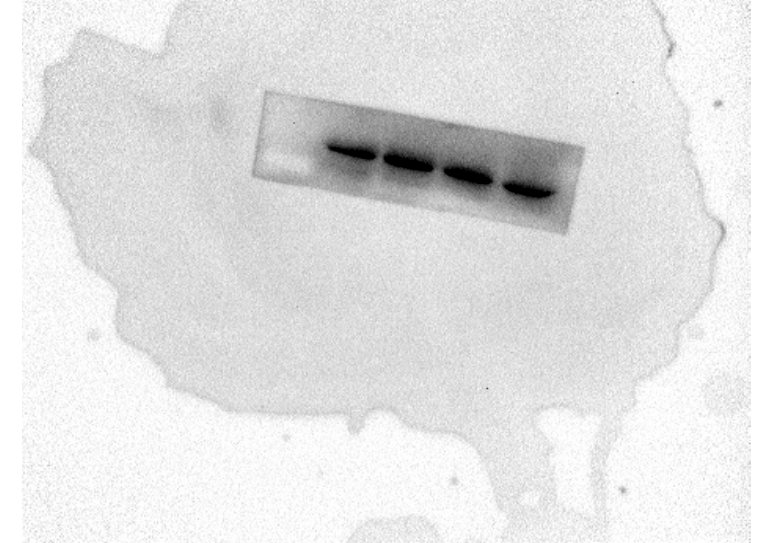

Supplement: Supplementary file 1 [file DataSheet_1.zip › WBs/6/6a/New folder/p-p65tu.tif]

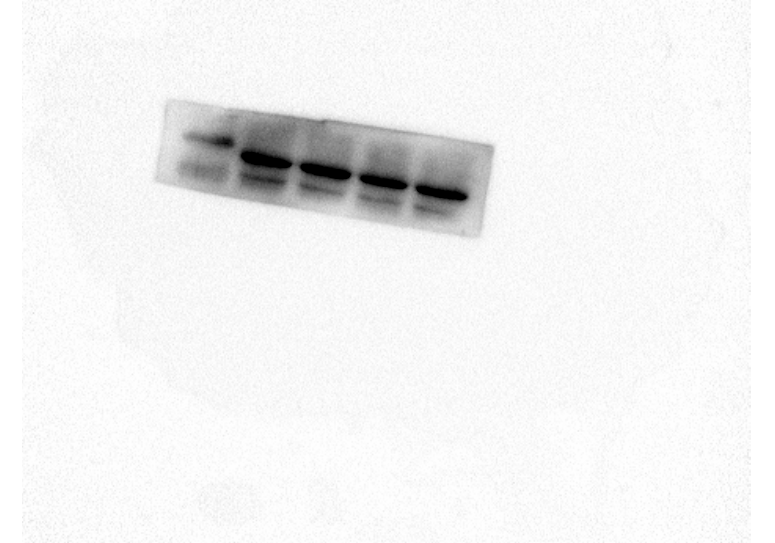

Supplement: Supplementary file 1 [file DataSheet_1.zip › WBs/6/6a/New folder/tp65.tif]

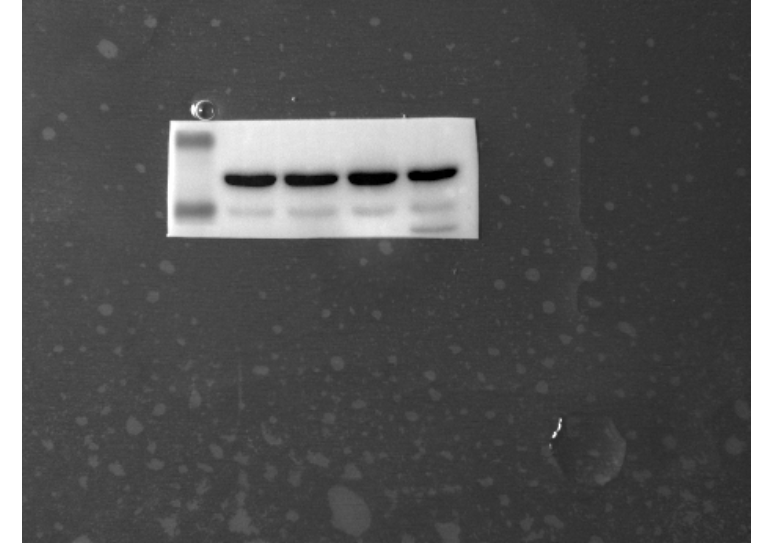

Supplement: Supplementary file 1 [file DataSheet_1.zip › WBs/6/6b/New folder/HE GD.tif]

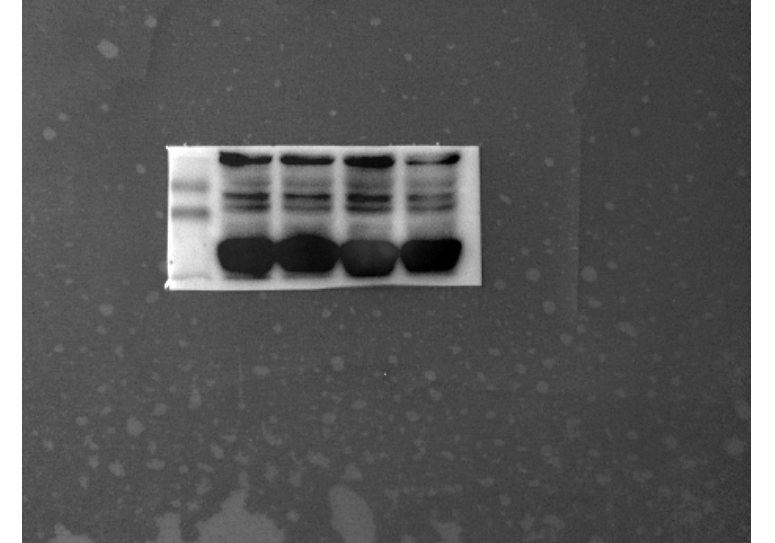

Supplement: Supplementary file 1 [file DataSheet_1.zip › WBs/6/6b/New folder/HE IKBA.tif]

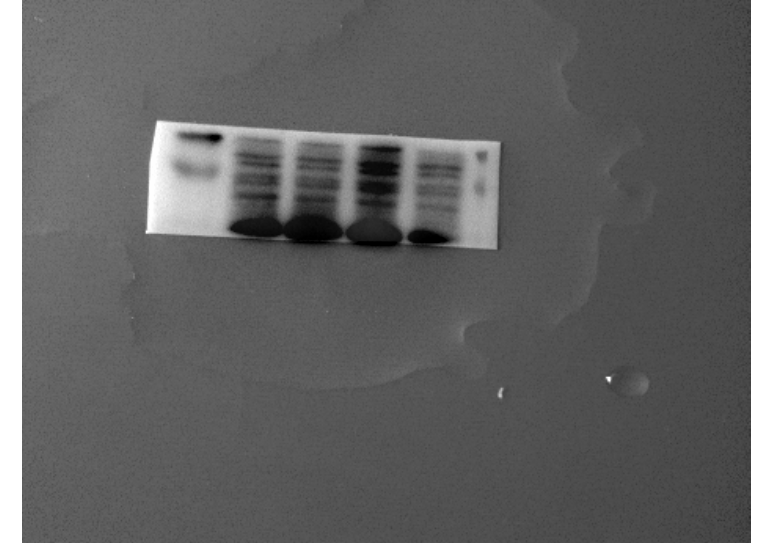

Supplement: Supplementary file 1 [file DataSheet_1.zip › WBs/6/6b/New folder/HE LCN2.tif]

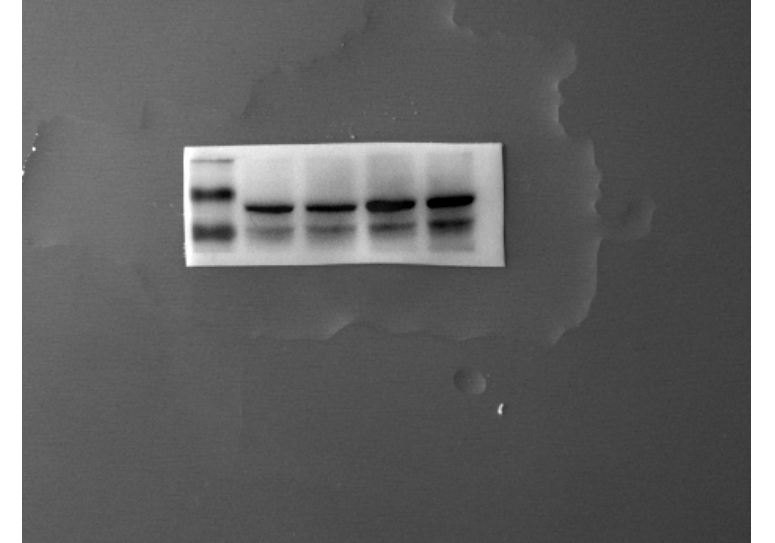

Supplement: Supplementary file 1 [file DataSheet_1.zip › WBs/6/6b/New folder/HE P65.tif]

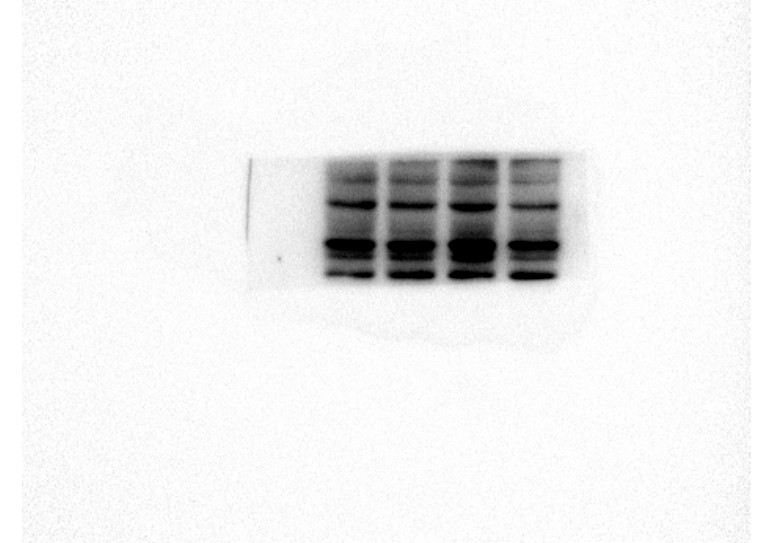

Supplement: Supplementary file 1 [file DataSheet_1.zip › WBs/6/6b/New folder/P-IKBA.tif]

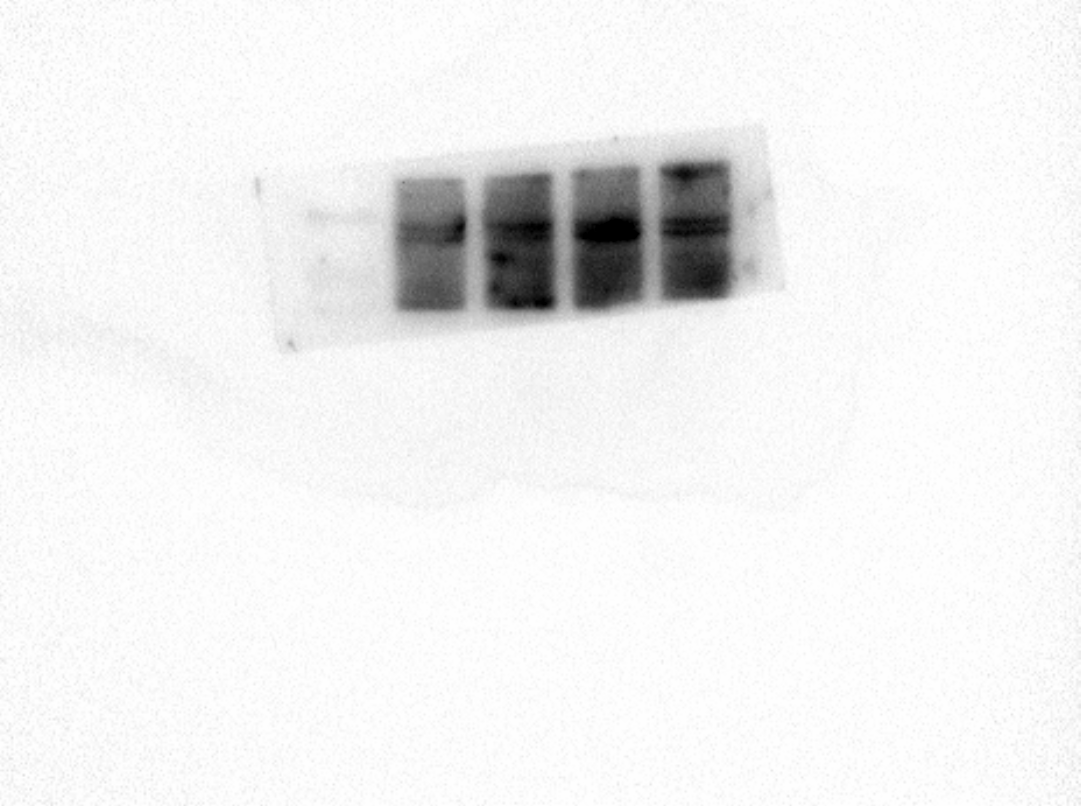

Supplement: Supplementary file 1 [file DataSheet_1.zip › WBs/6/6b/New folder/P-P65.tif]
