# Supplementary material for: Lipocalin 2 Is a Regulator During Macrophage Polarization Induced by Soluble Worm Antigens
Source: Front Cell Infect Microbiol. 2021 Sep 20;11:747135. doi: 10.3389/fcimb.2021.747135 (PMC8489661; doi:10.3389/fcimb.2021.747135)
Supplement: Supplementary file 2 [file DataSheet_2.pdf]

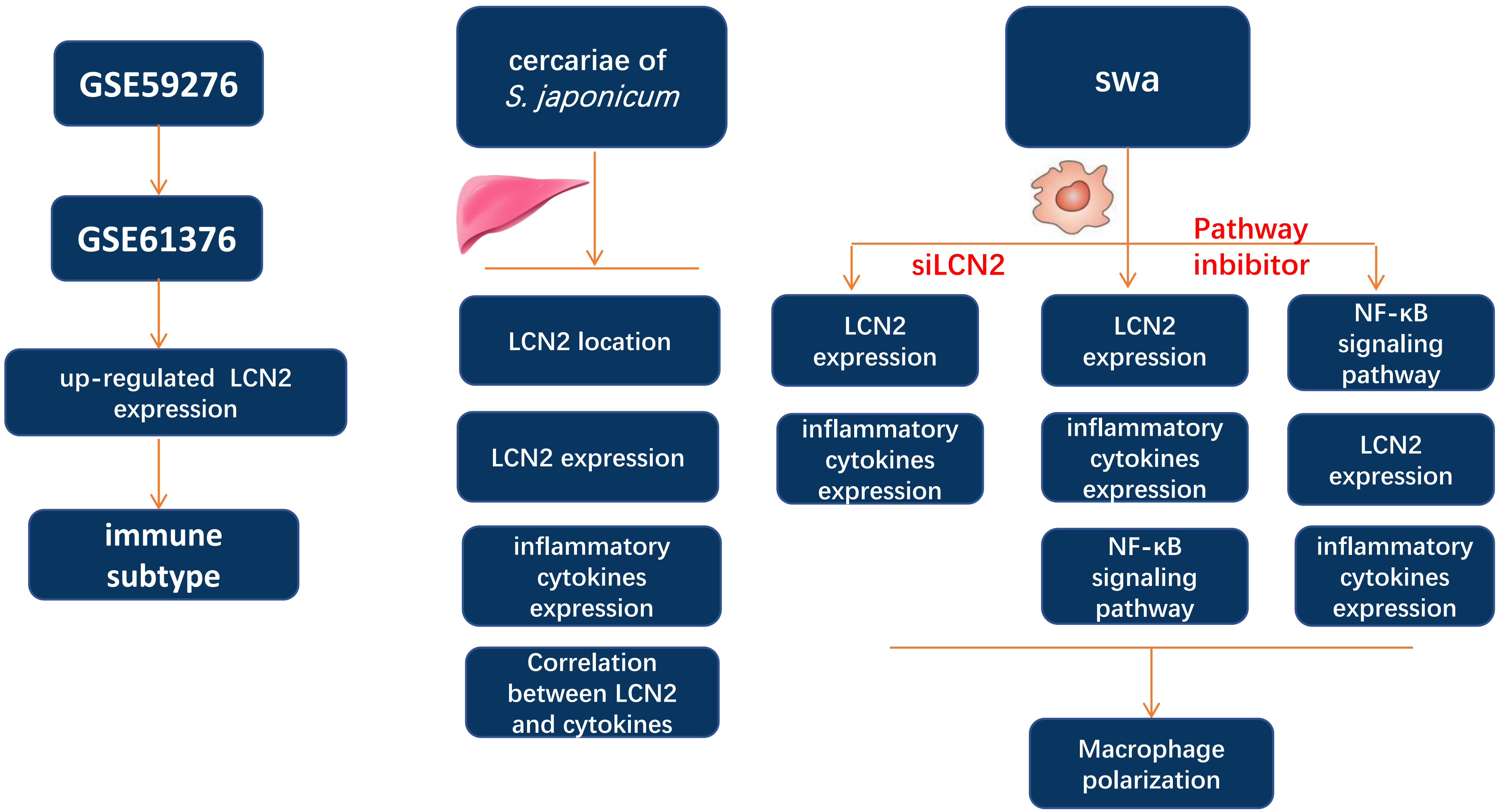
GSE59276
GSE61376
up-regulated LCN2 expression
immune subtype
cercariae of *S. japonicum*
LCN2 location
LCN2 expression
inflammatory cytokines expression
Correlation between LCN2 and cytokines
swa
siLCN2
Pathway inbibitor
LCN2 expression
inflammatory cytokines expression
LCN2 expression
inflammatory cytokines expression
NF-κB signaling pathway
NF-κB signaling pathway
LCN2 expression
inflammatory cytokines expression
Macrophage polarization

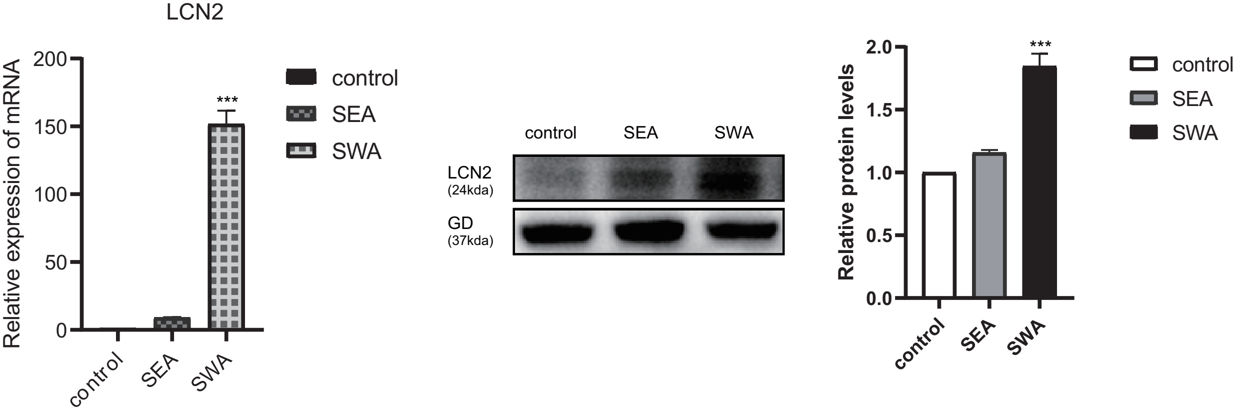

LCN2
Relative expression of mRNA
200
150
100
50
0
***
control
SEA
SWA
control
SEA
SWA
LCN2
(24kda)
GD
(37kda)
Relative protein levels
2.0
1.5
1.0
0.5
0.0
***
control
SEA
SWA
control
SEA
SWA
