# Supplementary material for: Lipocalin 2 Is a Regulator During Macrophage Polarization Induced by Soluble Worm Antigens
Source: Front Cell Infect Microbiol. 2021 Sep 20;11:747135. doi: 10.3389/fcimb.2021.747135 (PMC8489661; doi:10.3389/fcimb.2021.747135)
Supplement: Supplementary file 3 [file Table_1.docx]

**Sup Table1** 222 overlap DEGs between normal and *S. japonicum* via GSE59276

| row.names(tT) | logFC | AveExpr | t | P.Value | adj.P.Val | B |
| --- | --- | --- | --- | --- | --- | --- |
| LCN2 | 7.603753 | 2.175541 | 67.77226 | 1.43E-16 | 3.67E-12 | 26.43957 |
| S100A8 | 6.496042 | -0.43712 | 54.66944 | 1.76E-15 | 2.26E-11 | 24.82269 |
| S100A9 | 6.231862 | -0.44363 | 34.35156 | 3.95E-13 | 5.97E-10 | 20.48479 |
| SAA3 | 6.114568 | 2.872579 | 21.64723 | 8.16E-11 | 1.94E-08 | 15.42714 |
| SLPI | 5.375873 | 0.296135 | 17.77351 | 7.73E-10 | 9.34E-08 | 13.15983 |
| SAA1 | 4.95711 | 2.416927 | 25.702 | 1.13E-11 | 5.35E-09 | 17.36285 |
| SAA2 | 4.84692 | 2.293007 | 49.88787 | 5.12E-15 | 3.29E-11 | 24.05199 |
| PRTN3 | 4.688712 | 2.073532 | 43.68598 | 2.41E-14 | 8.83E-11 | 22.85539 |
| CXCL1 | 4.596052 | 0.716596 | 48.703 | 6.77E-15 | 3.48E-11 | 23.84201 |
| CAMP | 4.348785 | 0.263505 | 32.65363 | 7.11E-13 | 9.61E-10 | 19.95676 |
| CHI3L3 | 4.240396 | 1.359351 | 14.27689 | 9.06E-09 | 5.56E-07 | 10.62179 |
| TIMP1 | 4.11954 | 1.654639 | 8.186976 | 3.62E-06 | 5.73E-05 | 4.369561 |
| CD14 | 4.033581 | 2.059281 | 25.93688 | 1.02E-11 | 5.15E-09 | 17.46395 |
| FCRL3 | 3.89041 | 1.30627 | 35.01886 | 3.16E-13 | 5.07E-10 | 20.6829 |
| UBD | 3.845664 | 1.211943 | 16.14153 | 2.29E-09 | 2E-07 | 12.04412 |
| ORM2 | 3.834862 | 1.813707 | 31.03859 | 1.28E-12 | 1.26E-09 | 19.42012 |
| PRG2 | 3.792075 | 0.487152 | 38.50926 | 1.05E-13 | 2.69E-10 | 21.64108 |
| SPINK3 | 3.717116 | 1.563994 | 26.06944 | 9.63E-12 | 5.05E-09 | 17.52054 |
| HIST1H2AO | 3.71311 | 1.118623 | 35.13879 | 3.03E-13 | 5.07E-10 | 20.71796 |
| SERPINA3G | 3.674628 | 1.256731 | 21.45305 | 9.05E-11 | 2.11E-08 | 15.32434 |
| MARCO | 3.605463 | 1.971501 | 21.96186 | 6.92E-11 | 1.73E-08 | 15.59155 |
| II | 3.56832 | 0.736155 | 27.26279 | 7.36E-12 | 3.89E-09 | 17.99078 |
| HIST1H2AN | 3.521854 | 1.267621 | 23.08473 | 3.90E-11 | 1.15E-08 | 16.15752 |
| H2-AB1 | 3.506672 | 0.772701 | 25.71165 | 1.15E-11 | 5.45E-09 | 17.36488 |
| CTSG | 3.504174 | 1.811966 | 23.62355 | 3.00E-11 | 9.62E-09 | 16.41814 |
| HIST1H2AF | 3.501081 | 1.205091 | 24.66541 | 1.82E-11 | 6.69E-09 | 16.90327 |
| CD52 | 3.472973 | 0.90388 | 26.19557 | 9.11E-12 | 5.02E-09 | 17.57406 |
| 2010001M09RIK | 3.452163 | 0.626567 | 10.81968 | 1.89E-07 | 5.54E-06 | 7.444177 |
| H2-EB1 | 3.432548 | 0.901909 | 22.56872 | 5.06E-11 | 1.36E-08 | 15.90137 |
| LAPTM5 | 3.432239 | 0.875508 | 32.22568 | 8.28E-13 | 1.01E-09 | 19.81796 |
| LGALS3 | 3.389319 | 0.936235 | 34.17048 | 4.20E-13 | 5.99E-10 | 20.43014 |
| MT2 | 3.351219 | 0.521224 | 17.99117 | 6.73E-10 | 8.78E-08 | 13.30061 |
| NE | 3.338272 | 1.608905 | 31.76067 | 9.81E-13 | 1.10E-09 | 19.6644 |
| RETNLG | 3.318363 | 0.622741 | 14.03505 | 1.1E-08 | 6.47E-07 | 10.42418 |
| S100A11 | 3.305707 | 0.545036 | 25.50871 | 1.24E-11 | 5.48E-09 | 17.27882 |
| SPP1 | 3.269527 | 0.301986 | 16.13414 | 2.30E-09 | 2E-07 | 12.03882 |
| H2-AA | 3.255542 | 0.792256 | 14.64623 | 6.82E-09 | 4.56E-07 | 10.91745 |
| FPR-RS2 | 3.200638 | 1.612961 | 23.25186 | 3.59E-11 | 1.11E-08 | 16.23909 |
| H2-DMB1 | 3.1913 | 0.917043 | 17.61941 | 8.53E-10 | 9.91E-08 | 13.05907 |
| ANXA2 | 3.070332 | 0.511336 | 25.40272 | 1.30E-11 | 5.65E-09 | 17.23242 |
| PKM2 | 3.064855 | 1.03933 | 20.2637 | 1.74E-10 | 3.36E-08 | 14.67147 |
| LY6D | 3.06016 | 1.019396 | 10.72174 | 3.57E-07 | 8.6E-06 | 7.292772 |
| LY6C | 3.059376 | 0.852562 | 27.66903 | 4.84E-12 | 3.46E-09 | 18.17734 |
| CKB | 3.052572 | 0.528158 | 25.0634 | 1.52E-11 | 6.12E-09 | 17.08235 |
| HIST1H2AD | 3.045001 | 1.103832 | 31.47008 | 1.09E-12 | 1.17E-09 | 19.56696 |
| CXCL13 | 3.005336 | 1.59217 | 16.47832 | 1.82E-09 | 1.68E-07 | 12.28351 |
| CXCL9 | 2.996692 | 0.506624 | 16.03774 | 2.47E-09 | 2.1E-07 | 11.96934 |
| LTF | 2.987429 | 0.288922 | 26.88688 | 1.83E-05 | 0.000187 | 12.73925 |
| IQGAP1 | 2.980368 | 0.81963 | 21.11748 | 1.08E-10 | 2.4E-08 | 15.14423 |
| HIST1H2AK | 2.962845 | 1.04637 | 30.02037 | 1.88E-12 | 1.73E-09 | 19.06307 |
| ALOX5AP | 2.948868 | 1.282321 | 17.72419 | 7.97E-10 | 9.57E-08 | 13.12768 |
| FCER1G | 2.914449 | 1.267699 | 28.86523 | 2.97E-12 | 2.38E-09 | 18.6391 |
| LY86 | 2.837305 | 1.092674 | 27.38902 | 5.44E-12 | 3.78E-09 | 18.06572 |
| HBA-A1 | 2.831423 | -0.08904 | 4.531084 | 0.00073 | 0.004551 | -1.25147 |
| NGP | 2.829561 | 0.772839 | 28.4737 | 3.48E-12 | 2.70E-09 | 18.49059 |
| S100A6 | 2.817969 | 0.694744 | 16.26237 | 2.11E-09 | 1.87E-07 | 12.13058 |
| COTL1 | 2.815447 | 0.731367 | 26.48431 | 8.03E-12 | 4.59E-09 | 17.69544 |
| PLAC8 | 2.806002 | 1.398128 | 18.09616 | 6.30E-10 | 8.39E-08 | 13.36789 |
| BC049975 | 2.80273 | 1.18804 | 15.14438 | 4.69E-09 | 3.48E-07 | 11.30492 |
| HDC | 2.783205 | 0.993884 | 13.6265 | 1.52E-08 | 8.3E-07 | 10.08282 |
| LST1 | 2.774799 | 1.21604 | 29.41622 | 2.38E-12 | 2.07E-09 | 18.84392 |
| VCAM1 | 2.76777 | 0.885178 | 25.51849 | 1.23E-11 | 5.48E-09 | 17.28309 |
| IER3 | 2.742447 | 1.423858 | 8.319503 | 2.94E-06 | 0.000049 | 4.544502 |
| LY6A | 2.731873 | 0.216255 | 16.54529 | 1.74E-09 | 1.62E-07 | 12.33052 |
| APCS | 2.713316 | 1.171492 | 23.36474 | 3.40E-11 | 1.07E-08 | 16.29381 |
| HIST1H2AH | 2.698441 | 1.048498 | 28.09179 | 4.06E-12 | 2.98E-09 | 18.34328 |
| PRC1 | 2.672458 | 1.355224 | 15.53258 | 3.53E-09 | 2.81E-07 | 11.5983 |
| P2RY6 | 2.65643 | 0.962325 | 17.0746 | 1.22E-09 | 1.28E-07 | 12.6954 |
| GBP1 | 2.656106 | 1.249617 | 16.76137 | 1.50E-09 | 1.47E-07 | 12.48089 |
| HBB-B1 | 2.646354 | 0.538425 | 5.542804 | 0.000139 | 0.001132 | 0.483378 |
| EAR2 | 2.63867 | 1.055982 | 20.8304 | 1.27E-10 | 2.65E-08 | 14.98764 |
| CCL8 | 2.630853 | 1.446982 | 4.958774 | 0.000355 | 0.002507 | -0.50223 |
| GVIN1 | 2.60546 | 0.1133 | 22.5467 | 5.12E-11 | 1.36E-08 | 15.89029 |
| CYBA | 2.592003 | 1.145441 | 24.23161 | 2.24E-11 | 7.76E-09 | 16.7042 |
| SH3BGRL3 | 2.585454 | 0.798185 | 24.87082 | 1.66E-11 | 6.45E-09 | 16.99611 |
| ABI3 | 2.583602 | 0.88788 | 20.68833 | 1.37E-10 | 2.82E-08 | 14.90927 |
| C1QB | 2.574348 | 0.950991 | 20.97708 | 1.17E-10 | 2.53E-08 | 15.06794 |
| CHI3L1 | 2.573614 | 1.444586 | 15.77423 | 2.97E-09 | 2.43E-07 | 11.77727 |
| VAV1 | 2.572378 | 1.190852 | 25.75807 | 1.11E-11 | 5.35E-09 | 17.38708 |
| SDC3 | 2.550935 | 1.036255 | 21.2578 | 1.01E-10 | 2.3E-08 | 15.21993 |
| 0610041G09RIK | 2.548383 | 0.715405 | 7.546679 | 7.81E-06 | 0.000107 | 3.513672 |
| SERPINA1E | 2.541854 | 0.153876 | 3.805147 | 0.00261 | 0.012951 | -2.56621 |
| TGM2 | 2.511261 | 0.590186 | 22.74427 | 4.63E-11 | 1.28E-08 | 15.98925 |
| AIF1 | 2.481061 | 1.056459 | 17.52378 | 9.07E-10 | 1.04E-07 | 12.99608 |
| IL1B | 2.467559 | 1.150161 | 13.2649 | 2.05E-08 | 1.04E-06 | 9.772511 |
| LYZS | 2.464971 | 0.783912 | 16.33262 | 2.01E-09 | 1.81E-07 | 12.18055 |
| C1QG | 2.447728 | 0.871948 | 20.12181 | 1.88E-10 | 3.51E-08 | 14.5908 |
| PSCDBP | 2.438895 | 0.537881 | 13.32661 | 1.94E-08 | 9.98E-07 | 9.826026 |
| CXCL4 | 2.438294 | 1.320886 | 14.99381 | 5.25E-09 | 3.78E-07 | 11.18914 |
| HK3 | 2.417408 | 1.089959 | 23.99474 | 2.50E-11 | 8.46E-09 | 16.59376 |
| FXYD5 | 2.411871 | 0.733605 | 18.77883 | 4.14E-10 | 6.22E-08 | 13.79559 |
| TMSB4X | 2.405068 | 0.613866 | 24.73975 | 1.76E-11 | 6.65E-09 | 16.93697 |
| SPON2 | 2.402631 | 0.388302 | 8.937315 | 1.41E-06 | 2.72E-05 | 5.319338 |
| 2610001E17RIK | 2.39271 | 0.211185 | 14.33105 | 8.69E-09 | 5.42E-07 | 10.66561 |
| PFC | 2.371468 | 1.182441 | 17.91285 | 7.07E-10 | 9E-08 | 13.25016 |
| SLC4A1 | 2.371399 | 0.596888 | 16.89303 | 1.37E-09 | 1.4E-07 | 12.57155 |
| PGLYRP1 | 2.367201 | 0.98092 | 21.53264 | 8.68E-11 | 2.05E-08 | 15.3666 |
| MCM5 | 2.360235 | 1.11977 | 18.70736 | 4.32E-10 | 6.34E-08 | 13.75158 |
| NCF4 | 2.35929 | 1.03725 | 25.68097 | 1.14E-11 | 5.35E-09 | 17.35374 |
| GPX3 | 2.357774 | 0.20358 | 14.56546 | 7.25E-09 | 4.79E-07 | 10.85342 |
| LPL | 2.336732 | -0.2413 | 16.02338 | 2.49E-09 | 2.12E-07 | 11.95896 |
| LRG1 | 2.329679 | 0.446264 | 16.58695 | 1.69E-09 | 1.6E-07 | 12.35966 |
| EAR4 | 2.32141 | 1.061099 | 24.31762 | 2.15E-11 | 7.56E-09 | 16.744 |
| SLC15A3 | 2.317324 | 1.067332 | 17.519 | 9.10E-10 | 1.04E-07 | 12.99292 |
| C1QA | 2.306636 | 0.947596 | 21.68726 | 7.99E-11 | 1.92E-08 | 15.4482 |
| H2-DMA | 2.301081 | 0.857728 | 15.54755 | 3.49E-09 | 2.79E-07 | 11.60947 |
| D17H6S56E-5 | 2.281881 | 1.286778 | 7.743871 | 6.05E-06 | 8.71E-05 | 3.783506 |
| HK2 | 2.275398 | 0.978171 | 20.15489 | 1.85E-10 | 3.48E-08 | 14.60967 |
| IFITM6 | 2.272082 | 0.96753 | 12.78921 | 3.06E-08 | 1.41E-06 | 9.351991 |
| F13A1 | 2.27108 | 1.238187 | 18.18145 | 5.97E-10 | 8.16E-08 | 13.42224 |
| LGMN | 2.269939 | 0.75224 | 17.95058 | 6.90E-10 | 8.84E-08 | 13.27449 |
| UAP1L1 | 2.263839 | 0.568585 | 23.58 | 3.06E-11 | 9.71E-09 | 16.39733 |
| PIP5K2A | 2.225907 | 0.661448 | 21.99525 | 6.80E-11 | 1.71E-08 | 15.60884 |
| PSMB9 | 2.201808 | 0.244521 | 15.93505 | 2.65E-09 | 2.24E-07 | 11.89486 |
| LOXL1 | 2.194399 | 0.184274 | 5.43786 | 0.000164 | 0.001307 | 0.309635 |
| BAT2 | 2.184027 | -0.02153 | 22.77599 | 4.56E-11 | 1.27E-08 | 16.00504 |
| HEMP1 | 2.172889 | 0.713977 | 22.88744 | 4.31E-11 | 1.22E-08 | 16.06035 |
| CORO1A | 2.172655 | 0.642061 | 18.50591 | 8.38E-10 | 9.03E-08 | 13.57251 |
| CASP1 | 2.156258 | 0.648071 | 17.42977 | 9.64E-10 | 1.1E-07 | 12.93381 |
| 1200002N14RIK | 2.149897 | 1.035844 | 17.36841 | 1E-09 | 1.11E-07 | 12.89298 |
| AXL | 2.143949 | 0.702962 | 23.65955 | 2.94E-11 | 9.57E-09 | 16.43531 |
| PLEKHA2 | 2.143409 | 0.3384 | 16.63425 | 1.63E-09 | 1.57E-07 | 12.39267 |
| CIRBP | 2.12692 | 1.094309 | 15.31086 | 4.15E-09 | 3.16E-07 | 11.43164 |
| TMSB10 | 2.117481 | 0.509043 | 20.78832 | 1.30E-10 | 2.69E-08 | 14.96449 |
| FBN1 | 2.102965 | 0.516597 | 8.151494 | 3.62E-06 | 5.78E-05 | 4.326407 |
| SELL | 2.09759 | 1.082389 | 22.1105 | 6.40E-11 | 1.65E-08 | 15.66831 |
| ADRBK1 | 2.095332 | 0.793696 | 17.05908 | 1.23E-09 | 1.28E-07 | 12.68487 |
| COL4A1 | 2.091646 | 0.281463 | 11.23126 | 1.26E-07 | 4.05E-06 | 7.866977 |
| ISYNA1 | 2.090384 | 1.202969 | 10.85367 | 1.82E-07 | 5.43E-06 | 7.479623 |
| LOC100034251 | 2.089569 | 0.971356 | 15.3906 | 3.92E-09 | 3.05E-07 | 11.49185 |
| IFI30 | 2.08511 | 1.181001 | 20.65111 | 3.73E-09 | 2.47E-07 | 14.31978 |
| EMB | 2.077748 | 1.00788 | 16.5559 | 1.72E-09 | 1.62E-07 | 12.33794 |
| MYO1F | 2.069823 | 0.870786 | 16.86058 | 1.40E-09 | 1.41E-07 | 12.54927 |
| CLEC4N | 2.059213 | 1.08414 | 14.06127 | 1.07E-08 | 6.41E-07 | 10.44576 |
| TYROBP | 2.055162 | 1.085658 | 20.91605 | 1.21E-10 | 2.57E-08 | 15.03461 |
| EMR1 | 2.054722 | 0.991828 | 18.1173 | 6.22E-10 | 8.32E-08 | 13.38138 |
| 2310057H16RIK | 2.05167 | 0.902518 | 15.44924 | 3.75E-09 | 2.97E-07 | 11.53593 |
| SLC13A3 | 2.051185 | 0.177695 | 13.45841 | 1.56E-07 | 4.15E-06 | 9.640045 |
| CSRP1 | 2.043564 | 0.396062 | 19.60589 | 2.53E-10 | 4.43E-08 | 14.29223 |
| MS4A6D | 2.03921 | 0.992591 | 15.37157 | 9.42E-09 | 5.23E-07 | 11.38626 |
| CXCL16 | 2.012962 | 0.498265 | 16.12255 | 2.32E-09 | 2E-07 | 12.03048 |
| AADAC | -2.00066 | -0.09226 | -21.2483 | 1.01E-10 | 2.3E-08 | 15.2148 |
| 0610038K03RIK | -2.00634 | -0.72177 | -13.4676 | 1.73E-08 | 9.14E-07 | 9.947404 |
| PCK1 | -2.00639 | -0.24868 | -9.848 | 5.13E-07 | 1.22E-05 | 6.388861 |
| 1300013J15RIK | -2.00654 | -0.38618 | -17.6036 | 1.26E-09 | 1.24E-07 | 13.01204 |
| ABCA8A | -2.0216 | -0.32886 | -14.0785 | 1.06E-08 | 6.37E-07 | 10.45995 |
| SPP2 | -2.0216 | -0.74619 | -11.6192 | 8.73E-08 | 3.07E-06 | 8.253279 |
| MOD1 | -2.02829 | -0.31613 | -11.4454 | 1.07E-07 | 3.56E-06 | 8.078614 |
| OAT | -2.02862 | -0.67261 | -14.3356 | 8.66E-09 | 5.42E-07 | 10.6693 |
| ACAA2 | -2.03398 | -0.37452 | -15.1801 | 4.59E-09 | 3.4E-07 | 11.33196 |
| HSD11B1 | -2.05007 | -0.70392 | -12.1085 | 9.96E-08 | 3.19E-06 | 8.669964 |
| MUP2 | -2.05481 | -0.84268 | -14.3711 | 1.97E-08 | 9.18E-07 | 10.60912 |
| SDH1 | -2.05578 | -0.88333 | -18.5593 | 4.73E-10 | 6.71E-08 | 13.65988 |
| HAL | -2.05589 | -1.37325 | -14.0966 | 1.04E-08 | 6.31E-07 | 10.47482 |
| GSTT2 | -2.08636 | -0.74191 | -16.3078 | 2.04E-09 | 1.84E-07 | 12.16292 |
| 2310016A09RIK | -2.10291 | -0.87221 | -16.7583 | 1.50E-09 | 1.47E-07 | 12.47874 |
| DDT | -2.10583 | -0.56486 | -16.4744 | 1.82E-09 | 1.68E-07 | 12.28076 |
| CML1 | -2.17399 | -0.82904 | -22.9065 | 4.27E-11 | 1.22E-08 | 16.06976 |
| GSTA2 | -2.20524 | 0.165659 | -15.7905 | 2.94E-09 | 2.42E-07 | 11.78925 |
| EHHADH | -2.20853 | -0.53508 | -20.9273 | 1.20E-10 | 2.57E-08 | 15.04074 |
| ANGPTL4 | -2.23277 | 0.127033 | -8.11626 | 3.78E-06 | 5.99E-05 | 4.28026 |
| RAPGEF4 | -2.2593 | 0.295506 | -18.6 | 4.61E-10 | 6.66E-08 | 13.68513 |
| D0H4S114 | -2.25932 | -0.57651 | -4.98271 | 0.000342 | 0.002428 | -0.46096 |
| HAO3 | -2.27124 | 0.277609 | -13.6952 | 1.44E-08 | 7.95E-07 | 10.14086 |
| ABCB11 | -2.30497 | -1.01905 | -23.0894 | 3.90E-11 | 1.15E-08 | 16.1598 |
| 3110049J23RIK | -2.31412 | -1.14806 | -21.2122 | 1.03E-10 | 2.32E-08 | 15.1954 |
| IGFBP2 | -2.32101 | -0.29525 | -12.9134 | 7.01E-07 | 1.36E-05 | 8.945916 |
| CYP2D26 | -2.36199 | -0.38377 | -18.8918 | 3.86E-10 | 5.95E-08 | 13.86477 |
| DCI | -2.36204 | -0.74265 | -22.959 | 4.16E-11 | 1.2E-08 | 16.0957 |
| DHRS6 | -2.36643 | -0.96817 | -12.4521 | 4.1E-08 | 1.75E-06 | 9.045123 |
| ES22 | -2.37807 | -1.32727 | -17.3628 | 1.01E-09 | 1.11E-07 | 12.88926 |
| GNMT | -2.39454 | -1.09274 | -18.1165 | 3.98E-09 | 2.64E-07 | 13.13049 |
| LOC382044 | -2.40047 | -0.528 | -13.1039 | 2.34E-08 | 1.15E-06 | 9.631762 |
| EG13909 | -2.43711 | -0.9703 | -13.0522 | 2.45E-08 | 1.18E-06 | 9.586255 |
| AKR1C19 | -2.4429 | -0.91018 | -11.4209 | 1.05E-07 | 3.51E-06 | 8.057242 |
| SUCNR1 | -2.4561 | -1.12308 | -14.6782 | 6.65E-09 | 4.46E-07 | 10.94271 |
| MUP3 | -2.50303 | -1.45493 | -7.85546 | 5.24E-06 | 7.77E-05 | 3.934104 |
| ASL | -2.51623 | -0.48441 | -17.6725 | 1.05E-09 | 1.11E-07 | 13.07097 |
| 4732433M03 | -2.52825 | -1.00992 | -10.961 | 1.64E-07 | 4.99E-06 | 7.590851 |
| CYP2F2 | -2.53086 | -1.37581 | -14.7586 | 6.26E-09 | 4.29E-07 | 11.00595 |
| LOC620807 | -2.54058 | -0.99964 | -15.075 | 4.94E-09 | 3.6E-07 | 11.25171 |
| GLS2 | -2.55453 | -1.22799 | -17.8145 | 7.56E-10 | 9.32E-08 | 13.18612 |
| CYP2C50 | -2.57948 | -0.8954 | -12.6042 | 7.11E-07 | 1.37E-05 | 8.727631 |
| GSTT1 | -2.65636 | -1.17912 | -18.058 | 6.45E-10 | 8.49E-08 | 13.34346 |
| ABCG8 | -2.68067 | -0.72326 | -29.3762 | 2.42E-12 | 2.07E-09 | 18.82921 |
| CAT | -2.69886 | -0.49332 | -17.3742 | 9.99E-10 | 1.11E-07 | 12.89686 |
| 1300012D20RIK | -2.74021 | -0.74567 | -24.4873 | 1.98E-11 | 7.07E-09 | 16.82203 |
| CYP3A11 | -2.75908 | -1.03408 | -26.7137 | 7.26E-12 | 4.40E-09 | 17.79074 |
| UGT1A10 | -2.76077 | -0.67762 | -20.835 | 1.26E-10 | 2.65E-08 | 14.99018 |
| A1BG | -2.76682 | -1.64299 | -12.3659 | 4.43E-08 | 1.86E-06 | 8.965385 |
| AGXT2L1 | -2.78046 | -0.65816 | -25.6858 | 1.16E-11 | 5.46E-09 | 17.35425 |
| CYP3A25 | -2.90631 | -1.09105 | -19.119 | 3.37E-10 | 5.52E-08 | 14.0026 |
| RDH6 | -2.96218 | -1.10822 | -20.5023 | 1.52E-10 | 3.05E-08 | 14.80576 |
| G6PC | -2.98344 | -0.53631 | -17.8797 | 7.22E-10 | 9.05E-08 | 13.22876 |
| 1700018O18RIK | -3.00828 | -0.06935 | -8.88293 | 1.51E-06 | 2.87E-05 | 5.252791 |
| OTTMUSG00000007485 | -3.0935 | -1.13159 | -14.3212 | 8.75E-09 | 5.42E-07 | 10.65768 |
| DBP | -3.12542 | -0.44357 | -6.49062 | 3.33E-05 | 0.000348 | 1.984061 |
| CYP2B20 | -3.16016 | -0.05841 | -27.2046 | 5.89E-12 | 3.88E-09 | 17.99145 |
| MGC29978 | -3.17302 | -1.05099 | -22.5398 | 5.14E-11 | 1.36E-08 | 15.88683 |
| CPS1 | -3.29741 | -1.48378 | -14.1057 | 1.04E-08 | 6.28E-07 | 10.48227 |
| ALDH3A2 | -3.33998 | -0.973 | -30.957 | 1.32E-12 | 1.26E-09 | 19.39206 |
| THRSP | -3.3511 | -0.63825 | -5.06529 | 0.000298 | 0.002164 | -0.31912 |
| UPP2 | -3.46916 | -0.93978 | -9.35649 | 8.85E-07 | 1.88E-05 | 5.821203 |
| CYP2C37 | -3.51464 | -1.62319 | -7.82121 | 5.48E-06 | 8.05E-05 | 3.888041 |
| SERPINA6 | -3.55738 | -1.55312 | -12.8056 | 3.02E-08 | 1.4E-06 | 9.366675 |
| 0610039N19RIK | -3.57627 | -1.13439 | -25.2287 | 1.76E-11 | 6.79E-09 | 17.1339 |
| BC013476 | -3.67652 | -1.23428 | -24.1612 | 2.31E-11 | 7.92E-09 | 16.67149 |
| PTE2A | -3.76979 | -1.71036 | -37.179 | 1.57E-13 | 3.37E-10 | 21.29059 |
| GSTT3 | -3.78664 | -1.7144 | -19.0083 | 3.60E-10 | 5.75E-08 | 13.93571 |
| HAMP2 | -3.79387 | -1.69067 | -11.2405 | 1.25E-07 | 4.03E-06 | 7.876286 |
| KEG1 | -3.82997 | -1.22197 | -21.01 | 1.15E-10 | 2.5E-08 | 15.08587 |
| AU018778 | -3.89459 | -1.90848 | -22.4942 | 5.26E-11 | 1.38E-08 | 15.86383 |
| EG243881 | -4.03861 | -1.81599 | -38.8978 | 9.30E-14 | 2.66E-10 | 21.74025 |
| CYP8B1 | -4.32223 | -2.9526 | -11.1503 | 1.36E-07 | 4.32E-06 | 7.78485 |
| 2810007J24RIK | -4.33443 | -2.12336 | -37.2875 | 1.52E-13 | 3.37E-10 | 21.31984 |
| CYP2B9 | -4.38326 | -2.61894 | -24.6342 | 1.85E-11 | 6.69E-09 | 16.88907 |
| INMT | -4.5382 | -2.07721 | -24.8138 | 1.70E-11 | 6.52E-09 | 16.97043 |
| CYP2A5 | -4.87762 | -1.54564 | -18.6275 | 2.83E-08 | 1.11E-06 | 12.9183 |
| CYP4A14 | -5.07931 | -1.52108 | -25.9382 | 1.02E-11 | 5.15E-09 | 17.46452 |
| CAR3 | -5.09346 | -1.84756 | -41.0227 | 5.01E-14 | 1.61E-10 | 22.25858 |
| FMO3 | -5.18599 | -1.81759 | -19.4686 | 4.64E-10 | 6.08E-08 | 14.15833 |
| CYP2B13 | -5.83442 | -2.35267 | -51.2433 | 3.74E-15 | 3.21E-11 | 24.2825 |
