# Supplementary material for: Lipocalin 2 Is a Regulator During Macrophage Polarization Induced by Soluble Worm Antigens
Source: Front Cell Infect Microbiol. 2021 Sep 20;11:747135. doi: 10.3389/fcimb.2021.747135 (PMC8489661; doi:10.3389/fcimb.2021.747135)
Supplement: Supplementary file 4 [file Table_2.docx]

**Sup Table2** 22 types of infiltrated immune cells in normal and infection groups

| Mixture | GSM1432676 | GSM1432677 | GSM1432646 | GSM1432647 | GSM1432648 |
| --- | --- | --- | --- | --- | --- |
| B cells naive | 0.000 | 0.179 | 0.001 | 0.181 | 0.000 |
| B cells memory | 0.156 | 0.000 | 0.018 | 0.000 | 0.045 |
| Plasma cells | 0.000 | 0.061 | 0.000 | 0.000 | 0.000 |
| T cells CD8 | 0.001 | 0.000 | 0.000 | 0.016 | 0.000 |
| T cells CD4 naive | 0.134 | 0.000 | 0.059 | 0.065 | 0.117 |
| T cells CD4 memory resting | 0.000 | 0.125 | 0.000 | 0.000 | 0.000 |
| T cells CD4 memory activated | 0.330 | 0.034 | 0.000 | 0.000 | 0.000 |
| T cells follicular helper | 0.000 | 0.000 | 0.146 | 0.108 | 0.104 |
| T cells regulatory (Tregs) | 0.018 | 0.135 | 0.000 | 0.014 | 0.000 |
| T cells gamma delta | 0.000 | 0.000 | 0.109 | 0.015 | 0.053 |
| NK cells resting | 0.205 | 0.000 | 0.000 | 0.000 | 0.000 |
| NK cells activated | 0.000 | 0.085 | 0.012 | 0.022 | 0.014 |
| Monocytes | 0.032 | 0.183 | 0.098 | 0.000 | 0.292 |
| Macrophages M0 | 0.025 | 0.039 | 0.005 | 0.001 | 0.000 |
| Macrophages M1 | 0.000 | 0.000 | 0.117 | 0.056 | 0.051 |
| Macrophages M2 | 0.000 | 0.000 | 0.002 | 0.196 | 0.123 |
| Dendritic cells resting | 0.000 | 0.000 | 0.111 | 0.137 | 0.005 |
| Dendritic cells activated | 0.020 | 0.036 | 0.000 | 0.018 | 0.013 |
| Mast cells resting | 0.052 | 0.091 | 0.000 | 0.000 | 0.043 |
| Mast cells activated | 0.000 | 0.000 | 0.292 | 0.145 | 0.125 |
| Eosinophils | 0.000 | 0.031 | 0.029 | 0.000 | 0.016 |
| Neutrophils | 0.027 | 0.000 | 0.000 | 0.024 | 0.000 |
