# Supplementary material for: Lipocalin 2 Is a Regulator During Macrophage Polarization Induced by Soluble Worm Antigens
Source: Front Cell Infect Microbiol. 2021 Sep 20;11:747135. doi: 10.3389/fcimb.2021.747135 (PMC8489661; doi:10.3389/fcimb.2021.747135)
Supplement: Supplementary file 5 [file Table_3.docx]

Our raw data has been uploaded in https://www.jianguoyun.com/p/DSa5iggQyPTOCRjPtYIE.
